# Intrinsic, widefield optical imaging of hemodynamics in rodent models of Alzheimer’s disease and neurological injury

**DOI:** 10.1117/1.NPh.10.2.020601

**Published:** 2023-05-02

**Authors:** Christian Crouzet, Thinh Phan, Robert H. Wilson, Teo Jeon Shin, Bernard Choi

**Affiliations:** a University of California, Irvine, Beckman Laser Institute and Medical Clinic, Irvine, California, United States; b University of California, Irvine, Department of Biomedical Engineering, Irvine, California, United States; c University of California, Irvine, Department of Medicine, Irvine, California, United States; d Seoul National University, Department of Pediatric Dentistry and Dental Research Institute, Seoul, Republic of Korea; e University of California, Irvine, Department of Surgery, Irvine, California, United States; f University of California, Irvine, Edwards Lifesciences Foundation Cardiovascular Innovation Research Center, California, United States

**Keywords:** widefield optical imaging, spectroscopy, optical intrinsic signal imaging, laser speckle contrast imaging, spatial frequency domain imaging, metabolism

## Abstract

The complex cerebrovascular network is critical to controlling local cerebral blood flow (CBF) and maintaining brain homeostasis. Alzheimer’s disease (AD) and neurological injury can result in impaired CBF regulation, blood–brain barrier breakdown, neurovascular dysregulation, and ultimately impaired brain homeostasis. Measuring cortical hemodynamic changes in rodents can help elucidate the complex physiological dynamics that occur in AD and neurological injury. Widefield optical imaging approaches can measure hemodynamic information, such as CBF and oxygenation. These measurements can be performed over fields of view that range from millimeters to centimeters and probe up to the first few millimeters of rodent brain tissue. We discuss the principles and applications of three widefield optical imaging approaches that can measure cerebral hemodynamics: (1) optical intrinsic signal imaging, (2) laser speckle imaging, and (3) spatial frequency domain imaging. Future work in advancing widefield optical imaging approaches and employing multimodal instrumentation can enrich hemodynamic information content and help elucidate cerebrovascular mechanisms that lead to the development of therapeutic agents for AD and neurological injury.

## 1 Introduction

Although the brain is only 2% of the total body weight, it utilizes 20% of the heart’s cardiac output and 20% of the body’s oxygen and glucose supply under healthy conditions.^1^ To ensure the brain receives adequate blood flow and energy, the cerebrovascular network modulates its vessel diameter to control local cerebral blood flow (CBF) and maintain brain homeostasis.^2^^,^^3^ The cornerstone of these dynamics is the neurovascular unit (NVU). The NVU comprises different cell types, including endothelial cells, mural cells, glial cells, and neurons, which work together to regulate CBF and maintain the blood–brain barrier (BBB).^4^ However, neurodegeneration, which occurs in Alzheimer’s disease (AD) and neurological injury, can result in impaired CBF regulation, BBB breakdown, neurovascular dysregulation, and ultimately, impaired brain homeostasis.^2^^,^^5^ To investigate these dynamics in rodent models, studies have used microscopic, mesoscopic, and macroscopic imaging approaches.

Microscopic approaches, such as two-photon excitation fluorescence imaging, provide exquisite cellular resolution that can examine the interplay between individual cell-types of the NVU. These approaches typically perform measurements localized to the first $1\;{\mathrm{mm}}^{3}$ of cortical brain tissue. They can provide anatomical and physiological views of the vasculature, CBF, and oxygen tension, providing mechanistic information at the cellular level. However, these approaches are confined to a small field of view (FOV), making examining connectivity between different brain regions challenging. On the other end of the spectrum, macroscopic approaches, such as positron emission tomography and magnetic resonance imaging (MRI), can probe the entire brain and are commonly used clinically in humans. These approaches enable the segmentation and comparison between the dynamics of CBF, cerebral metabolic rate of oxygen (${\mathrm{CMRO}}_{2}$), and cerebral metabolic rate of glucose in different brain regions.^6^^–^^9^ However, macroscopic approaches are expensive and lack contrast mechanisms that simultaneously detect a wide range of functional parameters.^10^

Mesoscopic approaches interrogate spatial scales that fall between the microscopic and macroscopic levels. For example, widefield optical imaging is a mesoscopic approach with an FOV ranging from millimeters to centimeters and probes up to the first few millimeters of rodent brain tissue. Widefield optical imaging approaches have measured both hemodynamic and neural activity. In this review, we will focus on hemodynamic activity but great reviews discuss widefield optical imaging in the context of examining neural activity.^11^^–^^13^ Widefield optical imaging approaches use different sources of contrast that enable the measurement of multiple hemodynamic parameters, such as CBF, oxygenation, and metabolism. Furthermore, measuring the entire cortical surface enables multiple FOVs to be compared, including different brain regions and vascular compartments. These advantages are critical to the advancement and implementation of widefield optical imaging technologies to monitor cerebral hemodynamic information.

This review first gives an anatomical and physiological overview of the cerebrovasculature in rodents. Next, we describe the principles of three widefield optical imaging approaches that can measure cerebral hemodynamics: (1) optical intrinsic signal imaging (OISI), (2) laser speckle imaging (LSI), and (3) spatial frequency domain imaging (SFDI). Then, we discuss measurements that can be performed with these approaches, such as resting-state dynamics, neurovascular coupling (NVC), and cerebrovascular reactivity (CVR). Next, we discuss how each technology has been used to measure physiological changes in AD and neurological injuries. Finally, we discuss the future directions for widefield optical imaging technologies in measuring cerebral hemodynamics.

## 2 Cerebrovascular System

The cerebrovasculature delivers nutrients, such as oxygen and glucose, to the brain. These nutrients enter the surrounding brain tissue through the BBB, primarily in capillaries. Similarly, metabolic waste products, such as carbon dioxide (${\mathrm{CO}}_{2}$), cross the BBB into the bloodstream to be cleared from the body through the lungs, kidneys, and liver. Oxygen and ${\mathrm{CO}}_{2}$ rapidly diffuse across the BBB. Larger nutrients and waste products cross the BBB using regulated transport receptors.^14^ The importance of these basic cerebral functions is exemplified by the fact that the brain receives 20% of the cardiac output and consumes 20% of the body’s supply of oxygen and glucose.^15^ This high metabolic activity is facilitated by the large cerebral microvascular blood volume fraction (∼16% in mice).^16^

Blood flow to the mouse brain originates from the internal carotid and vertebral arteries.^17^ In contrast to the circle of Willis in humans, in mice, the cerebral arteries of Willis do not form a complete loop.^18^ Instead, two distinct domains are supplied by either the internal carotid or the basilar artery. Due to the significant role that these large arteries play in modulating cerebrovascular resistance,^19^ they have a prominent role in the overall regulation of CBF. From the circle of Willis, three main arteries (anterior, middle, and posterior cerebral arteries) arise that progressively branch into smaller pial arteries that are present on the surface of the brain. The pial vessels then plunge into the brain as penetrating arterioles that subsequently branch into precapillary arterioles and capillaries, which are critical in nutrient delivery and waste removal.

To efficiently deliver nutrients to and remove waste products from the brain, a process known as neurovascular coupling (NVC) occurs. In response to increased local neural activity, CBF rapidly increases,^20^^,^^21^ which brings nutrients (i.e., oxygen and glucose) to meet the increased metabolic demand. The supply is large enough to ensure that neurons distal to individual cerebral blood vessels receive the necessary metabolites via diffusion along a concentration gradient.^22^ Local control of CBF is modulated by the cells that comprise the NVU: endothelial cells, pericytes, vascular smooth muscle cells (VSMCs), astrocytes, microglia, and neurons.^5^^,^^20^^,^^23^^,^^24^ More specifically, as local neuronal activity increases, surrounding arterioles vasodilate. An increase in local CBF occurs as a retrograde signaling cascade propagates to upstream arteries, including the pial arteries.^25^^–^^27^ Gap junctions facilitate rapid retrograde propagation, which is proposed to occur via K+ ions released during neural activity,^28^ of the signal between endothelial cells,^29^ and surrounding pericytes and VSMCs subsequently relax, leading to local vasodilatation. Vasoactive mediators, such as nitric oxide, epoxyeicosatrienoic acids (EETs), and prostaglandin E2 (PGE2), act in concert to achieve vasodilation during NVC.^28^^,^^30^ Interneurons may also modulate vessel diameter.^31^^,^^32^

In addition to NVC, cerebrovascular health and function can be studied via CVR tests to external stimuli. For example, pharmacological procedures can be performed to induce local vasodilatation, including superfusion of acetylcholine, which causes the release of endothelial nitric oxide^33^ and administration of nitric oxide donors (i.e., S-nitroso-N-acetylpenicillamine) to the exposed mouse cortex.^34^ Another commonly used approach to study CVR is hypercapnia, which uses increased inspired ${\mathrm{CO}}_{2}$ to stimulate vasodilation.^35^ In contrast, the superfusion of amyloid-beta (Aβ) leads to impaired CVR.^35^ To quantify CVR, CBF responses to these external stimuli are typically measured using optical technologies, such as laser Doppler flowmetry^34^^,^^35^ and LSI,^36^^,^^37^ and medical imaging technologies such as blood-oxygen-level-dependent functional MRI (fMRI)^38^ is used.

The general NVU composition varies at different levels of the cerebral vasculature.^2^^,^^28^ Cerebral arteries contain multiple layers of VSMCs to control CBF. Pial arterioles on the cortical surface have a thinner layer of VSMCs. In contrast, penetrating arterioles have a single VSMC layer that adjoins the endothelial cells. A perivascular space separates the basement membrane of these arterioles from the endfeet of astrocytes. Macrophages and leptomeningeal cells surround these arterioles. With smaller intraparenchymal arterioles, the perivascular space is no longer present, and astrocytes directly connect with the basement membrane. As penetrating arterioles branch into smaller intraparenchymal arterioles, transitional pericytes may be present with properties similar to both pericytes and VSMCs. Pericytes replace VSMCs at the capillary level, and neurons are close to a vascular basement membrane that consists of both pericytes and endothelial cells. Similar to arterioles, venules have a perivascular space that separates the vascular basement membrane from astrocytes. Larger veins have a thinner and flatter layer of VSMCs than arteries and a progressively larger perivascular space separating the vascular basement membrane from astrocytes. Schaeffer and Iadecola^28^ provide an excellent description of what they call the neurovascular complex, which consists of multiple versions of the NVU along the length of the cerebrovasculature. For example, they summarize RNA sequencing work that identified seven endothelial cell clusters in the NVU. These local variations in NVU enable efficient and robust communication throughout the cerebral vasculature and cerebrovascular dynamics throughout the entire brain.

The arteries and arterioles also play a significant role in regulating CBF in response to changes in arterial pressure. The pulsatility of blood due to the beating heart is dampened by the arteries and arterioles, resulting in minimal pulsatility at the capillary level. Also, cerebral autoregulation processes maintain a stable CBF over a wide range of arterial blood pressures. The myogenic response of VSMCs enables vasoconstriction at high arterial pressure and vasodilation at low arterial pressure, to achieve sufficient (but not excessive) CBF for different intravascular pressures.^39^

## 3 Core Technologies

### 3.1 Sources of Contrast

OISI, LSI, and SFDI use sources of optical contrast that are critical to measuring hemodynamics: (1) optical absorption, (2) optical scattering, and (3) speckle contrast. Since each source of contrast varies depending on the wavelength, care should be taken when selecting the wavelength(s) of light for device builds.

Absorption occurs when a chromophore (e.g., oxyhemoglobin) absorbs a photon of light. Scattering, which is high in biological tissue at visible and near-infrared wavelengths, occurs when a photon changes direction due to local refractive index mismatches. The absorption coefficient ($\mu_{a}$) is defined as the probability of absorption per unit distance, and the scattering coefficient ($\mu_{s}$) is defined as the probability of scattering per unit distance. The absorption due to hemoglobin is essential to measure cerebral hemodynamics. Visible to near-infrared (400 to 1000 nm) wavelengths have a highly variable absorption spectrum. The wavelengths from ∼400 to 600 nm provide excellent signal-to-noise to measure hemoglobin changes, whereas wavelengths from ∼650 to 1000 nm allow for increased depth penetration due to the near-infrared optical window. The near-infrared optical window exists due to an ∼100-fold decrease in hemoglobin absorption from visible to near-infrared light and is commonly used in diffuse optics.

The reduced scattering coefficient ($\mu_{s}^{\prime }$) is commonly used in place of $\mu_{s}$ to describe tissue optical properties. It comprises $\mu_{s}$ and anisotropy (g) of the tissue, where $\mu_{s}^{\prime }=(1-g)\mu_{s}$. g is the average cosine of all scattering angles and describes how forward-scattering each scattering event is. A typical value for g is ∼0.9 in biological tissue. $\mu_{s}^{\prime }$ is used in the diffuse regime (i.e., $\mu_{a}\ll \mu_{s}^{\prime }$) and describes the propagation of photons experiencing multiple scattering events. Unlike absorption, which is highly variable in the visible to near-infrared regime, scattering decreases with increasing wavelength as a power law function.

Speckle contrast is the last source of optical contrast we discuss. It can be obtained from analyzing the speckle pattern resulting from coherent light interference. The speckle contrast describes how much the speckle pattern fluctuates over time. The faster the speckle pattern fluctuates, the lower the speckle contrast, whereas the slower the speckle pattern fluctuates, the higher the speckle contrast. This information is used to obtain quantitative blood flow information. The three sources of optical contrast (absorption, scattering, and speckle contrast) are critical and instrumental for OISI, LSI, and SFDI to provide cerebral hemodynamic information. Next, we discuss the basic principles associated with each imaging approach.

### 3.2 Optical Intrinsic Signal Imaging

**Fig. 1:**
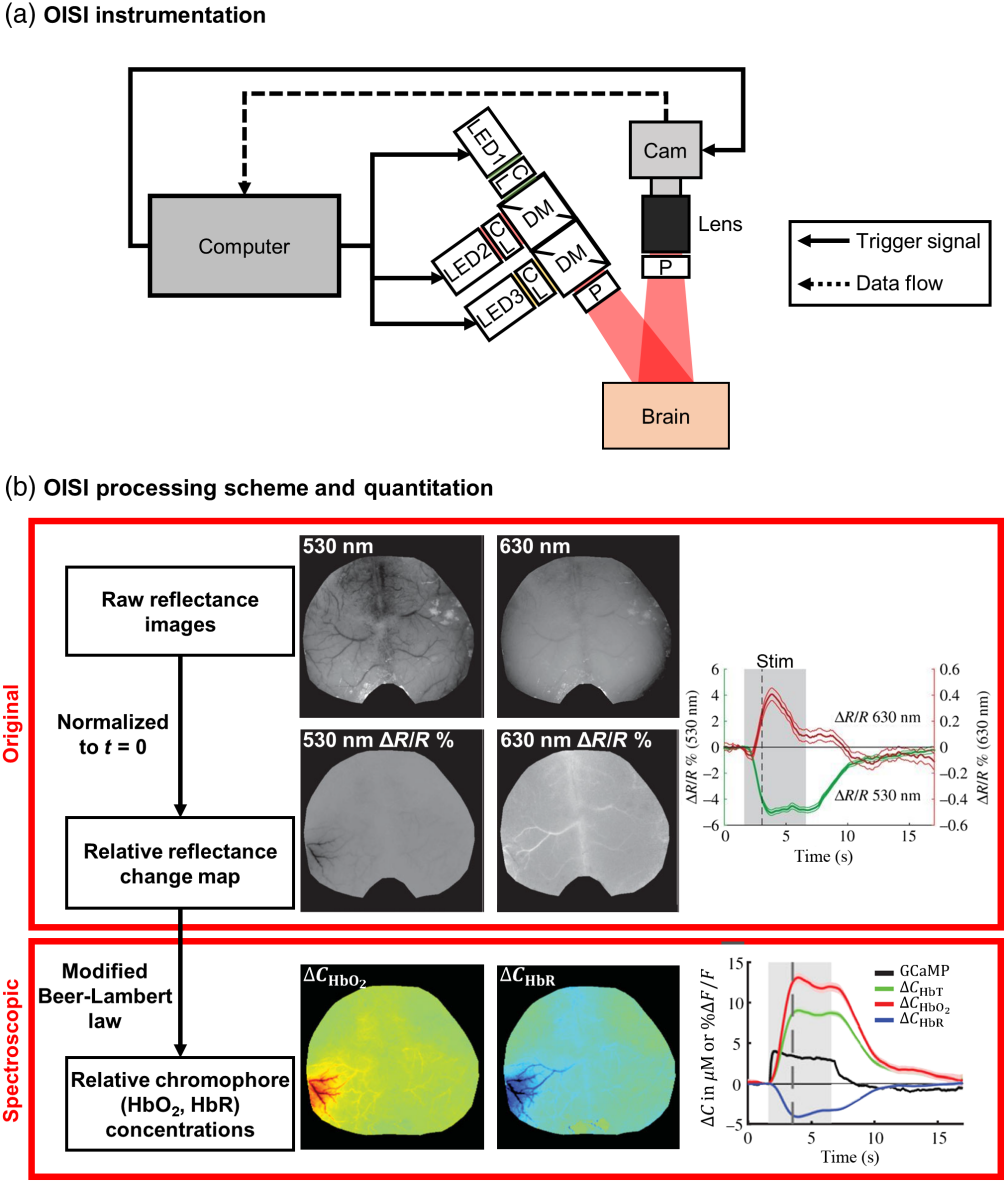
OISI instrumentation, processing scheme, and data quantification. (a) A typical OISI setup where a computer syncs the exposure time of the camera and switching of the LEDs. DM, dichroic mirror; CL, condenser lens; P, polarizer; LED, light-emitting diode; Cam, camera. (b) OISI processing pipeline from raw data with representative images of whisker stimulation on awake mice (adapted from Ref. 12). (Top) Using the original OISI approach, relative reflectance changes in % of 530 (∼HbT) and 630 nm (∼HbR) light during whisker stimulation are visualized (adapted from Ref. 12). (Bottom) Using spectroscopic OISI, relative changes in $C_{\mathrm{HbR}}$, $C_{\mathrm{HbO}2}$, $C_{\mathrm{HbT}}$, and calcium indicator GCaMP fluorescence during whisker stimulation are visualized (adapted from Ref. 12).

OISI or intrinsic signal optical imaging was developed by Grinvald et al.^40^ to visualize and quantify information related to neural activity. OISI measures changes in the reflected light from tissue associated with absorption and scattering dynamics. As the name suggests, OISI does not require using exogenous probes typically required for extrinsic fluorescence (e.g., calcium or voltage-sensitive dyes) or phosphorescence (e.g., oxygen tracing). The basic principle of OISI is that a perturbation can induce a change in reflectance (measured with a camera or sensor array) that gives a map of neural activities.^40^ To assess these dynamics, a typical hardware setup for OISI includes a camera sensor, a light source with one or more wavelengths, and a computer for triggering, acquisition, and processing [Fig. 1(a)].^12^^,^^41^^–^^43^ Light sources for OISI can use light-emitting diodes (LEDs) or a broadband light source (such as a lamp) with a filtered wheel.^43^

Since its conception, OISI has been widely used in neuroimaging due to its ability to provide high spatial and temporal resolution maps of brain activity. The depth sensitivity of OISI is typically localized to the cortical layers, often through a cranial window preparation. In rodents, the procedure often entails a craniectomy to expose the cortical region of interest (ROI), followed by a glass coverslip sealant. OISI has also been used with thinned^44^^,^^45^ and intact skull preparations on mice.^41^^,^^46^^,^^47^ Nevertheless, all methods require the retraction of the scalp and removal of the fascia layer before imaging.

The original method for OISI uses reflectance changes from the backscattered light to observe the spatiotemporal dynamics of functional activity [Fig. 1(b), top].^40^ A key improvement involves spectroscopic analysis [Fig. 1(b), bottom], which enables the measurement of changes in chromophores, such as oxyhemoglobin (${\mathrm{HbO}}_{2}$) and deoxyhemoglobin (HbR).^48^ Spectroscopic OISI employs the modified Beer–Lambert law [Eq. (1)]:^49^^,^^50^

$$I=I_{0}*\exp(-\mu_{a}*X+G),$$ (1)

where I is the detected light intensity, $I_{0}$ is the light source intensity, X is the total path length of light in tissue, $\mu_{a}$ is the absorption coefficient, and G is a geometry factor.^49^ It should be noted that X=(DPF)x, where DPF is the differential path length, which is a scaling factor between total path length and the source–detector separation, x.^12^ In near-infrared spectroscopy, the DPF is often used instead of the total path length since the source–detector separation is a known constant.^51^ Next, spectroscopic OISI often describes results to be relative to the initial time, $t_{0}$. Thus, Eq. (1) can be rewritten as

$$\Delta \mu_{a}(t,\lambda )=\mu_{a}(t,\lambda )-\mu_{a}(t_{0},\lambda )=-\frac{1}{X(\lambda )}\;\ln(\frac{I(t,\lambda )}{I(t_{0},\lambda )}).$$ (2)

The complete derivation for Eqs. (2)–(4) is described in Ref. 12. If a minimum of two wavelengths ($\lambda_{1}$ and $\lambda_{2}$) are used, and $\mu_{a}$ is defined as the sum of products between the chromophore concentrations of interest (i.e., ${\mathrm{HbO}}_{2}$ and HbR) and their associated extinction coefficients (ε), the following set of equations are obtained:

$$\Delta \mu_{a}(t,\lambda_{1})=\varepsilon_{{\mathrm{HbO}}_{2}}(\lambda_{1})\Delta C_{{\mathrm{HbO}}_{2}}(t)+\varepsilon_{Hb}(\lambda_{1})\Delta C_{\mathrm{HbR}}(t)\;\Delta \mu_{a}(t,\lambda_{2})=\varepsilon_{{\mathrm{HbO}}_{2}}(\lambda_{2})\Delta C_{{\mathrm{HbO}}_{2}}(t)+\varepsilon_{\mathrm{Hb}}(\lambda_{2})\Delta C_{\mathrm{HbR}}(t).$$ (3)

For Eqs. (2) and (3), X is required to estimate the change in concentrations of ${\mathrm{HbO}}_{2}$ ($\Delta C_{\mathrm{HbO}2}$) and HbR ($\Delta C_{\mathrm{HbR}}$). In recent studies, Monte Carlo simulations of photon migration in homogeneous media have been utilized to obtain estimates of X.^44^^,^^52^ To run the simulation, researchers often use brain optical properties ($\mu_{a}$ and $\mu_{s}^{\prime }$) from the literature.^53^ Using literature values of X, one can then achieve a wavelength-dependent linear system of equations with chromophore concentrations as unknowns. Theoretically, the system of equations can be solved algebraically for N chromophores using N wavelengths. However, a more common practice involves using least square fitting for the matrix form of the equations. This method requires at least N+1 wavelengths to improve fitting accuracy:

$$[\begin{array}{c} \Delta \mu_{a}(t,\lambda_{1})\\ \vdots \\ \Delta \mu_{a}(t,\lambda_{n}) \end{array}]=[\begin{array}{cc} \varepsilon_{{\mathrm{HbO}}_{2}}(\lambda_{1}) & \varepsilon_{\mathrm{HbR}}(\lambda_{1})\\ \vdots & \vdots \\ \varepsilon_{{\mathrm{HbO}}_{2}}(\lambda_{n}) & \varepsilon_{\mathrm{HbR}}(\lambda_{n}) \end{array}][\begin{array}{c} \Delta C_{{\mathrm{HbO}}_{2}}(t)\\ \Delta C_{\mathrm{HbR}}(t) \end{array}].$$ (4)

Using the above principles, OISI can measure absorption dynamics due to changes in blood volume, CBF, and tissue oxygenation in response to neuronal activation. In addition, OISI can also detect a change in reflectance associated with a change in scattering properties, which may be due to vascular and extracellular compartment changes, neuronal cellular expansion, and movement of water and ions.^54^^,^^55^ Although OISI performs well at measuring relative changes in hemoglobin concentrations, it does not measure absolute hemoglobin concentrations well due to the inability of OISI to separate effects due to absorption and scattering. We discuss how SFDI overcomes this limitation later in this review.

### 3.3 Laser Speckle Imaging

**Fig. 2:**
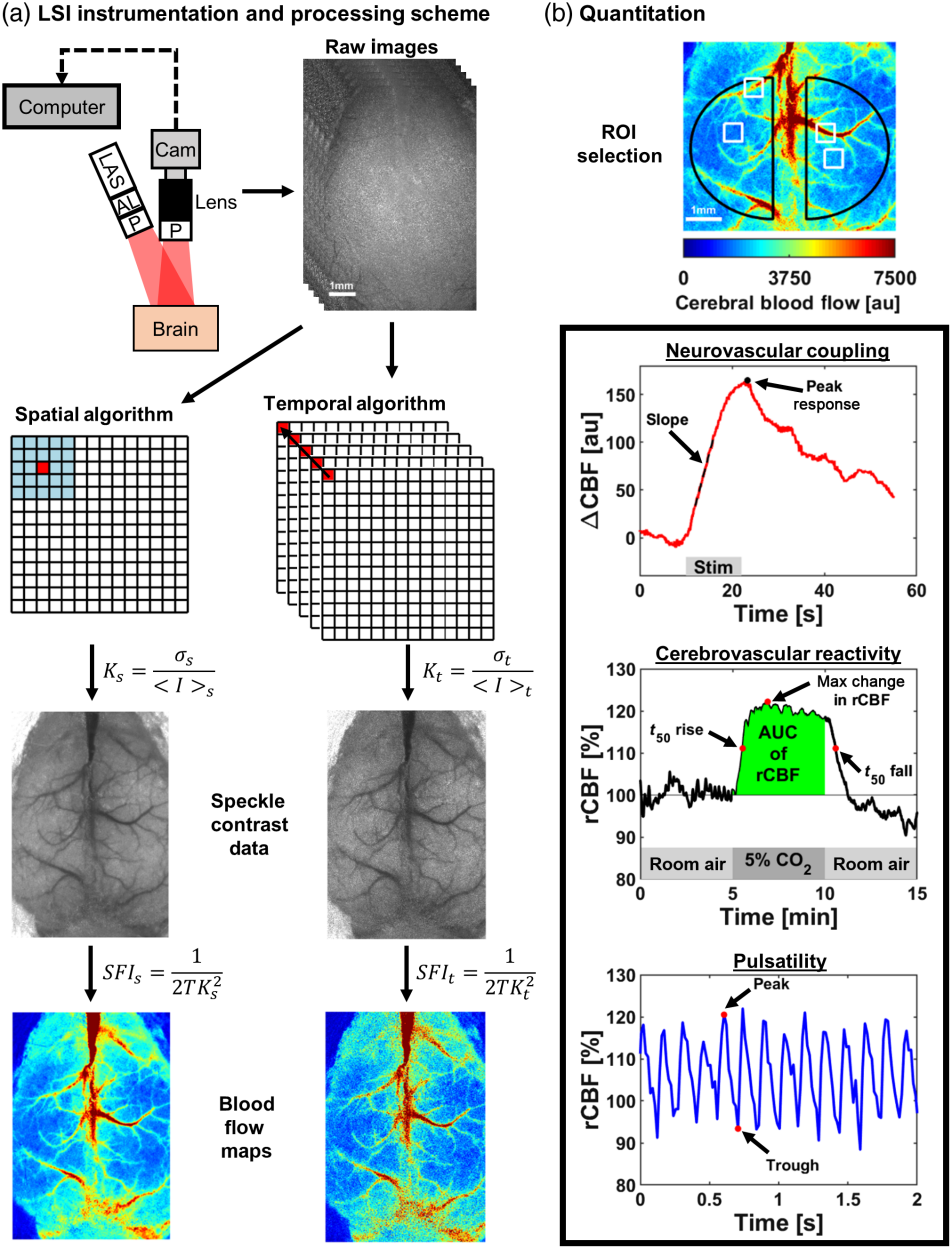
LSI instrumentation, processing scheme, and data quantification. (a) A typical LSI setup where a computer controls the camera. LAS, laser; AL, aspheric lens; P, polarizer; and Cam, camera. LSI processing pipeline from raw data to blood flow maps using (left) spatial and (right) temporal processing algorithms. (b) Quantitation of LSI data. Choosing an ROI depends on the brain being investigated. Three different types of measurements: (top) NVC in response to hindpaw stimulation with quantitative parameters, slope, and peak response; (middle) CVR in response to ${\mathrm{CO}}_{2}$ inhalation with quantitative parameters, $t_{50\;\text{rise}}$, AUC of relative CBF during hypercapnia, maximum change in relative CBF (rCBF), and $t_{50\;\text{fall}}$; (bottom) CBF pulsatility at rest with peak and trough outlined, showing an amplitude can be measured.

LSI, also known as laser speckle contrast imaging (LSCI) and laser speckle contrast analysis (LASCA), is a simple widefield imaging approach to measure relative blood flow. Fercher and Briers demonstrated the first use of LSI to visualize blood flow in biological tissue.^56^ Twenty years after this initial demonstration, Dunn et al.^57^ applied LSI to the brain and visualized CBF dynamics during cortical spreading depression (CSD). Since the publication of this seminal work, LSI has been applied to several neurologically relevant applications, such as stroke,^58^^,^^59^ cardiac arrest,^60^^–^^62^ AD,^63^ and aging^64^ to assess CBF dynamics. To obtain blood flow information, LSI hardware requires a laser, camera, sample to image, and computer for data acquisition and analysis [Fig. 2(a)]. When coherent laser light illuminates a sample, a random interference pattern, called a speckle pattern, is produced on the camera sensor. The speckle pattern contains bright and dark pixel intensities due to light that travels different path lengths in the sample. The bright and dark pixel intensities on the camera sensor are locations where constructive and destructive interference occur, respectively. When scattering particles (e.g., RBCs) move in a sample, the speckle pattern fluctuates in time, which appears as intensity variations on the camera detector. These intensity variations can be used to obtain information regarding the motion (e.g., blood flow) of the scattering particles.

An outline of traditional LSI data processing is shown in Fig. 2(a). A raw speckle image comprises a map of intensity fluctuations integrated over the camera’s exposure time (typically 1 to 10 ms for LSI). In areas of increased blood flow, the intensity fluctuations of the speckle pattern change more quickly, leading to a blurring of the raw speckle image. To quantify blood flow from the speckle pattern fluctuations, researchers have primarily used two processing algorithms to account for the spatial and temporal statistics of the speckle pattern. The spatial algorithm typically uses a 5×5 or 7×7 sliding window. The spatial standard deviation ($\sigma_{s}$) and spatial mean (${\left\langle I\right\rangle }_{s}$) are computed using the pixel intensities within the sliding window and assign the center pixel the spatial speckle contrast ($K_{s}$). The sliding window slides throughout the entire image until the computation is complete. The temporal algorithm computes the temporal speckle contrast ($K_{t}$) from a series of images. The temporal standard deviation ($\sigma_{t}$) and temporal mean (${\left\langle I\right\rangle }_{t}$) are calculated using the pixel intensities at each pixel location across a series of images. From the calculated speckle contrast, spatial or temporal, a relative blood flow can be calculated by taking the inverse of the correlation time ($\tau_{c}$) in Eq. (5) (i.e., blood flow $\propto 1/\tau_{c}$). T is the exposure time of the camera and β is a normalization factor to account for speckle averaging due to a mismatch of speckle size and detector size, polarization, and coherence effects. β is normally assumed to be one but can be measured experimentally to obtain a β-corrected speckle contrast, which we discuss later in this section:

$$K=\frac{\sigma }{\left\langle I\right\rangle }={\left[\beta (\frac{\tau_{c}}{T}+\frac{\tau_{c}^{2}}{2T^{2}}(\exp(-\frac{2T}{\tau_{c}})-1))\right]}^{1/2}.$$ (5)

Our group and others have derived a simplified speckle imaging equation for calculating relative blood flow by imposing assumptions on Eqs. (5) and (6).^65^^,^^66^ The speckle flow index (SFI) is proportional to blood flow and approximates $\tau_{c}$ as $2{\mathrm{TK}}^{2}$:

$$\mathrm{SFI}=\frac{\beta }{2TK^{2}}.$$ (6)

There are advantages and disadvantages when using the spatial and temporal algorithms to obtain speckle contrast. For example, the spatial algorithm has a higher temporal resolution but lower spatial resolution than the temporal algorithm. Importantly, due to the increased temporal resolution with the spatial algorithm, it can resolve pulsatile fluctuations in blood flow with each contraction of the heart.^62^^,^^67^^–^^70^ Due to the principle of ergodicity, the spatial and temporal algorithms can approach the same quantitative result when a craniotomy is performed since the static scattering from the skull is removed. However, biological tissue still has static and dynamic scattering components, which makes it difficult to achieve ergodicity in biological tissue.^71^ Despite this, areas measured directly over a vessel may be ergodic if the scattering events only interact with that vessel. When the skull remains intact, the spatial algorithm produces an offset to the spatial speckle contrast due to the static scattering from the skull. The temporal algorithm is less susceptible to artifacts caused by the skull and minimally affects the temporal speckle contrast. Although the spatial and temporal algorithms can be used individually, spatiotemporal approaches that combine aspects of the spatial and temporal algorithms are commonly used.^72^ We discuss some of the limitations associated with LSI in the resting-state blood flow section and potential ways to overcome these limitations. Despite some of the limitations associated with traditional LSI, it has been extensively used in diseased and healthy rodent brains. In this review, we highlight areas that LSI has been applied.

### 3.4 Spatial Frequency Domain Imaging

**Fig. 3:**
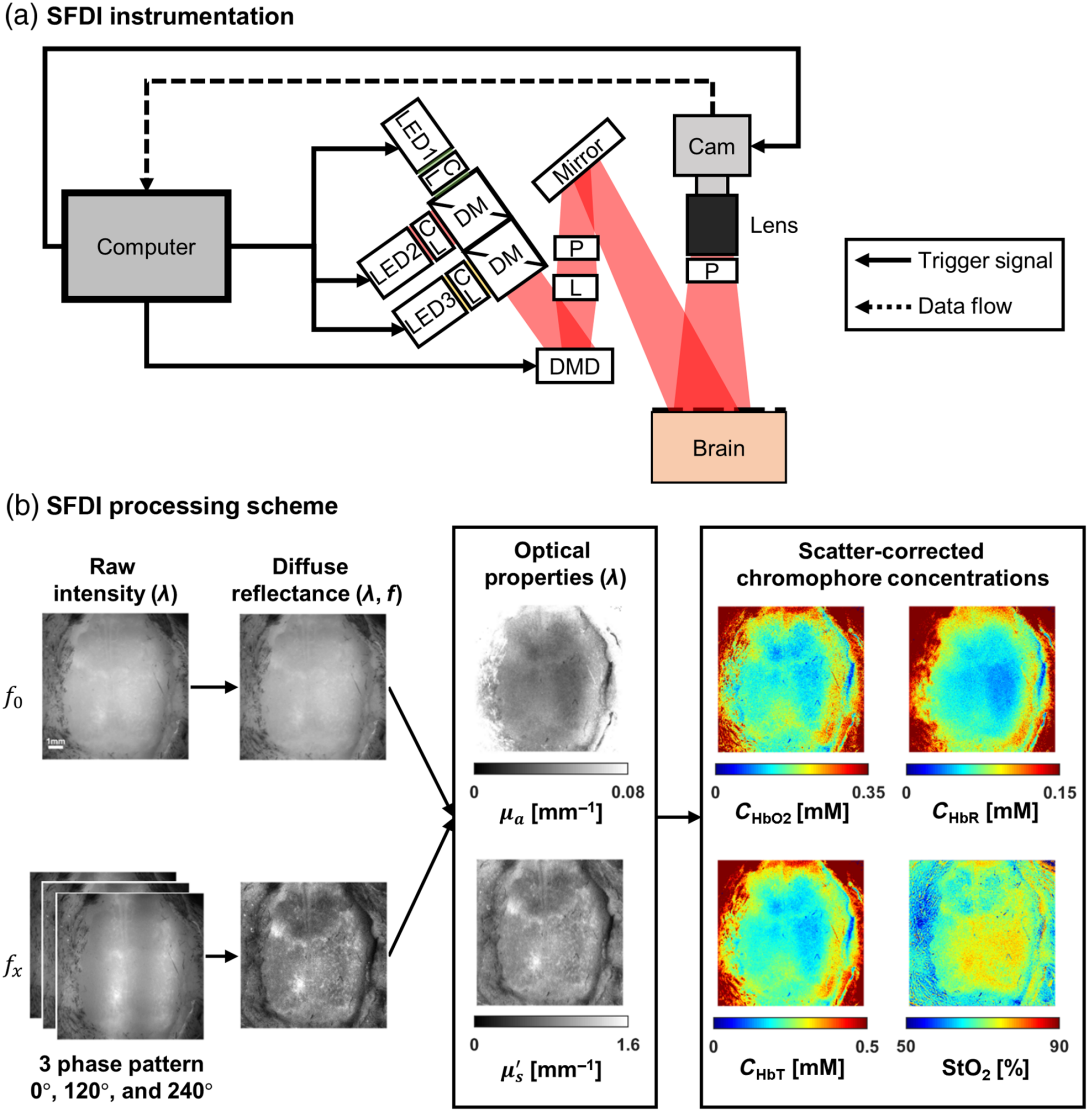
SFDI instrumentation and processing scheme. (a) A typical SFDI setup where a computer controls the camera, LEDs, and DMD. LED, light-emitting diode; CL, condenser lens; DM, dichroic mirror; DMD, digital micromirror device; P, polarizer; L, lens; and Cam, camera. (b) SFDI processing pipeline from raw data to tissue optical property maps and scatter-corrected chromophore concentrations. Raw intensity images at each wavelength are collected at two or more spatial frequencies, $f_{0}$ and $f_{x}$. Images with each $f_{x}$ are taken at three different phases (0 deg, 120 deg, and 240 deg) of the spatially modulated pattern. Next, the diffuse reflectance is calculated at each spatial frequency and wavelength. Using light-transport models, the optical properties ($\mu_{a}$ and $\mu_{s}^{\prime }$) as a function of wavelength λ are obtained. The scatter-corrected absorption can then be used to obtain chromophore concentrations (i.e., $C_{\mathrm{HbO}2}$, $C_{\mathrm{HbR}}$, $C_{\mathrm{HbT}}$) and tissue oxygenation (${\mathrm{StO}}_{2}$).

SFDI^73^ is a relatively new technology designed to quantitatively map tissue absorption and scattering over a wide FOV.^74^ To perform this measurement, a typical hardware setup for SFDI includes a camera sensor, a light source with one or more wavelengths, a digital micromirror device (DMD), and a computer for triggering, acquisition, and processing [Fig. 3(a)].^75^^–^^77^ SFDI uses a DMD to project spatially modulated patterns (either from a broadband source or multiple discrete wavelengths) onto the tissue and detects the backscattered light with a camera. The projected light patterns are typically sinusoidal, with different spatial frequencies. However, square-wave patterns have also been used in place of sinusoids to enable more rapid data acquisition.^78^ Each pattern projected onto the tissue undergoes amplitude attenuation and blurring due to tissue absorption and scattering, respectively. Since each spatial frequency has a unique combination of attenuation and blurring during its interaction with the tissue, using multiple different spatial frequencies enables the separation of the tissue $\mu_{a}$ from $\mu_{s}^{\prime }$.

Figure 3(b) shows the SFDI processing pipeline from raw data to tissue optical property maps and scatter-corrected chromophore concentrations. To process the raw SFDI data, the images collected at each spatial frequency are first demodulated to obtain the demodulated intensity image $I_{\mathrm{AC},\text{tissue}}$:

$$I_{\mathrm{AC},\text{tissue}}(f_{x})=\frac{\sqrt{2}}{3}[{\left(I_{1}-I_{2}\right)}^{2}+{\left(I_{2}-I_{3}\right)}^{2}+{\left(I_{3}-I_{1}\right)}^{2}],$$ (7)

where $I_{1}$, $I_{2}$, and $I_{3}$ are the two-dimensional raw backscattered intensity maps collected with three different spatial phase shifts of the SFDI pattern (typically 0, 120 deg, and 240 deg). The outputs from the demodulated images are the measured AC component of the reflectance ($I_{\mathrm{AC},\text{tissue}}(f_{x})$) maps at each spatial frequency. SFDI measurements also usually include an unstructured ($f_{x}=0$) backscattered intensity image (which does not require demodulation since it does not contain a spatial pattern). Next, the demodulated images are calibrated against a corresponding measurement $I_{\mathrm{AC},\text{phantom}}(f_{x})$ of a tissue-simulating phantom with known optical properties, using Eq. (8):

$$R_{d_{\text{tissue}}}(f_{x})=[R_{d_{\text{phantom}}}(f_{x})][\frac{I_{\mathrm{AC},\text{tissue}}(f_{x})}{I_{\mathrm{AC},\text{phantom}}(f_{x})}].$$ (8)

The resulting quantity $R_{d_{\text{tissue}}}(f_{x})$ is the diffuse reflectance of the tissue for each spatial frequency $f_{x}$ of the incident light. $R_{d_{\mathrm{tissue}}}(f_{x})$ is an intrinsic property of the tissue, independent of the components of the measurement setup, that varies from 0 (for a completely absorbing medium) to 1 (for a medium with no absorption or scattering-related losses). Two-dimensional maps of $R_{d_{\text{tissue}}}(f_{x})$ over the camera’s FOV are typically obtained at multiple wavelengths. Note that obtaining a quantitatively accurate value of $R_{d_{\text{tissue}}}(f_{x})$ depends on knowing $R_{d_{\text{phantom}}}(f_{x})$, which is typically obtained using a separate paradigm, such as using an integrating sphere.^79^

At each wavelength, the $R_{d_{\text{tissue}}}(f_{x})$ curve is fit with a mathematical model of light transport in tissue as a function of spatial frequency and tissue optical properties. This model is typically based on Monte Carlo simulations or the diffusion approximation to the radiative transport equation. The best fit of the model to $R_{d_{\text{tissue}}}(f_{x})$ at each wavelength is used to extract the corresponding $\mu_{a}$ and $\mu_{s}^{\prime }$ values at that wavelength. These spectrally resolved $\mu_{a}$ and $\mu_{s}^{\prime }$ values are fit to physiological models of tissue absorption and scattering to extract concentrations of different tissue components. For instance, in the case of absorption in brain tissue, the $\mu_{a}$ spectrum can be approximated as a linear combination of the molar extinction coefficients of oxygenated and deoxygenated hemoglobin, shown in Eq. (9):

$$\mu_{a}(\lambda )=2.303[\varepsilon_{\mathrm{HbO}2}(\lambda )C_{\mathrm{HbO}2}+\varepsilon_{\mathrm{HbR}}(\lambda )C_{\mathrm{HbR}}].$$ (9)

As stated in the OISI section, $\varepsilon_{{\mathrm{HbO}}_{2}}$ and $\varepsilon_{\mathrm{HbR}}$ are the molar extinction coefficients of oxygenated and deoxygenated hemoglobin, respectively, weighted by the concentrations of ${\mathrm{HbO}}_{2}$ and HbR. Thus, the best fit of this model to the $\mu_{a}(\lambda )$ spectrum yields the respective concentrations of ${\mathrm{HbO}}_{2}$ ($C_{\mathrm{HbO}2}$) and HbR ($C_{\mathrm{HbR}}$) of oxygenated and deoxygenated hemoglobin in the tissue. The total hemoglobin concentration ($C_{\mathrm{HbT}}$) of the tissue is thus given by the summation $C_{\mathrm{HbT}}=C_{\mathrm{HbO}2}+C_{\mathrm{HbR}}$, and the tissue oxygenation (${\mathrm{StO}}_{2}$) is given by the equation ${\mathrm{StO}}_{2}=100(C_{\mathrm{HbO}2}/C_{\mathrm{HbT}})$.

In addition, since illumination at different spatial frequencies is the frequency-domain analog of diffuse reflectance measurements at multiple spatial source–detector separations,^80^ SFDI effectively interrogates a volume of tissue whose depth depends on the spatial frequency. This feature of SFDI enables the separation of the contributions of different tissue layers to the detected signal.^81^ It can facilitate three-dimensional tomographic reconstruction of subsurface perturbations to tissue optical properties.^81^^,^^82^ Studies have also shown that using a combination of different orientations of structured light patterns^83^ or employing a wider range of spatial frequencies^84^ can provide sensitivity to higher-order parameters related to the directionality of the scattering of light by the tissue.

## 4 Types of Measurements

We discuss three measurements that widefield optical imaging approaches can perform: (1) resting-state, (2) NVC, and (3) CVR. These three measurements are used to assess cerebrovascular health and function. We discuss these measurements in the context of awake and anesthetized rodents.

### 4.1 Resting-State

Baseline or resting-state measurements can be used to examine unperturbed changes in hemodynamics in the awake or anesthetized state. Here, we highlight blood flow and functional connectivity as areas where widefield optical imaging approaches have significantly progressed and how quantitative information can be obtained from these areas.

#### 4.1.1 Blood flow

Traditional LSI is only able to measure relative blood flow changes accurately. Resting-state blood flow has been challenging to measure due to factors that influence the speckle contrast, such as day-to-day instrument variability,^85^ static scattering of the skull,^85^^,^^86^ and tissue optical properties.^87^^,^^88^ Equation (5) in the LSI section shows the direct relationship between the speckle contrast (K) and the decorrelation time ($\tau_{c}$,) where it is accepted that $\mathrm{CBF}∼1/\tau_{c}$.^85^^,^^86^^,^^89^ However, β in Eqs. (5) and (6) is a term that depends on the differences in day-to-day instrument variability, experimental environment, differences in detector-to-speckle size ratio, and polarization. However, the β term is normally assumed to be one, which does not take into account day-to-day instrument variability. To account for day-to-day instrument variability, β can be measured on each imaging day. Although β is fairly easy to measure, it is rarely performed. β can be measured by imaging an object where $\tau_{c}\gg T$, which is generally true for a static phantom. The measured β can be inserted into Eqs. (5) or (6) to obtain a more accurate blood flow measurement than assuming a β of one. This approach theoretically enables a comparison between animals and imaging days if the optical properties of the samples are similar.

Parthasarathy et al.^85^ implemented multiexposure speckle imaging (MESI) by deriving a new speckle equation and imaging system in the presence of static scattering (e.g., skull) that accounts for β, the fraction of dynamically scattered light, ergodicity of the sample, and noise of the imaging system. This methodology enables comparison across several days of imaging. Furthermore, MESI is theoretically more sensitive to blood flow changes than traditional LSI due to the multiple exposure times used. Although the technique works well, it has not gained widespread use, most likely due to the complexity of implementing the MESI system correctly compared with traditional LSI. Recently, an all-fiber-based illumination system was developed to reduce the size and complexity of traditional MESI systems.^90^ This design showed good agreement with standard MESI and may increase the adoption of MESI.^90^ In addition to MESI, advances have been made to better understand and improve the mathematical models used to describe laser speckle fluctuations in dynamic light scattering, including LSI. Postnov et al.^91^ performed a thorough characterization to investigate the type of motion (i.e., unordered or ordered) and scattering regime (i.e., single or multiple) used in dynamic light scattering. Importantly, this study revealed that the type of model used to obtain blood flow can vary widely over an FOV, especially for different vessel sizes, and can drastically impact the resultant blood flow measurement.^91^ Specifically, LSI can underestimate the decrease in CBF associated with ischemic stroke.^91^ A subsequent study^92^ further expanded on this work by deriving new models to be used directly for LSI that account for different combinations of scattering and motion type. Using the incorrect model can lead to errors of 300%.^92^ In addition to issues with modeling dynamic light scattering, optical properties also impact speckle contrast.^88^ To account for tissue optical properties in the speckle contrast, we have combined LSI with SFDI to measure tissue optical properties.^77^^,^^87^^,^^93^ The measured optical properties enable an optical property-corrected blood flow.^77^^,^^93^ Implementing these methods can lead to greater utility of LSI in longitudinal brain imaging studies.

When measuring resting-state CBF, it is important to consider the effects of anesthesia. Most inhaled anesthetics increase CBF. One example is isoflurane, an inhaled anesthetic commonly used during rodent surgeries and optical imaging. CBF dramatically increases via vasodilation under isoflurane anesthesia^94^ compared with the awake state, in addition to impairing cerebral autoregulation,^95^^–^^97^ and can lead to a diffuse NVC response. Alpha-chloralose is another common anesthetic used to assess NVC and cerebral autoregulation since both are preserved. However, resting-state CBF is drastically reduced in rodents that receive alpha-chloralose as an anesthetic.^98^^–^^100^ In addition, alpha-chloralose is a terminal anesthetic, which is not approved for survival experiments.^101^ Finally, ketamine-xylazine is a common anesthetic given i.p. in rodents to assess NVC and cerebral autoregulation. In comparison to isoflurane and alpha-chloralose, ketamine-xylazine minimally alters resting-state CBF.^98^ In all, anesthetics exert variable effects on resting-state CBF that differ from awake, resting-state CBF. If possible, resting-state CBF measurements with LSI should be made under awake conditions.

#### 4.1.2 Functional connectivity

Functional connectivity is the temporal correlation of neuronal activity between anatomically separated brain regions.^102^ The concept stems from image analysis using fMRI scans during rest, thus named resting-state functional connectivity. The presence of correlation during the resting state suggests that studies of its activities can be achieved without perturbations, such as stimulations or cognitive tasks.^41^ Widefield optical imaging approaches have enabled the analysis of functional connectivity in mice.^41^^,^^103^ OISI was the first widefield imaging technique used to obtain resting-state correlation maps over the mouse cortex.^41^ The method typically entails choosing a small ROI within a functional region of the brain (e.g., visual or motor) on the resulting hemoglobin concentration maps (e.g., ${\mathrm{HbO}}_{2}$ and HbT) as a seed pixel to obtain a time-traced signal.^41^^,^^46^^,^^103^ In addition, individual OISI wavelengths have been selected to perform functional connectivity analyses and are quantitatively comparable to ${\mathrm{HbO}}_{2}$ and HbT analyses.^104^ LSI was also used to obtain time-traced signals based on blood flow maps but has not seen much use in functional connectivity due to its inherent noisy nature and requires both spatial and temporal filtering.^103^ It is common practice to filter the time traces for the specific frequency bands of interest during the pixelwise time trace extraction, including infraslow (0.009 to 0.08 Hz), intermediate (0.08 to 0.4 Hz), and high (0.4 to 4.0 Hz) frequency bands.^46^ From these filtered time traces, a temporal correlation analysis is performed in a pairwise manner between a seed pixel and all other pixels over the sampled brain region. The correlation analysis yields a map of correlation coefficients (or fisher Z scores) relative to the seed pixel, which is the functional connectivity map.

When performing functional connectivity analysis, one important feature is the type of anesthesia used. For example, a study that used a transgenic mouse model with the calcium indicator, GCaMP6, showed that the spatial extent of seed-based functional connectivity vastly differs depending on the state of consciousness (i.e., awake versus anesthetized).^46^ Unfortunately, previous functional connectivity studies have used various anesthetic agents, which makes it difficult to compare findings. White et al. selected ketamine-xylazine as its anesthetic for OISI and showed it is appropriate to analyze signals in the infraslow band (0.009 to 0.08 Hz).^41^ In a study of functional connectivity with fMRI in rats, isoflurane reduced cross-correlation coefficients in comparison to alpha-chloralose and medetomidine.^105^ However, a subsequent fMRI study in mice showed that interhemispheric functional connectivity was diminished for alpha-chloralose and urethane in comparison to isoflurane and awake groups. Despite these studies, a recent study compared the effects of five different anesthetics: avertin, ketamine-xylazine, urethane, chloral hydrate, and isoflurane.^104^ This study showed that avertin and ketamine-xylazine produce robust and repeatable resting-state functional connectivity results, whereas isoflurane and chloral hydrate did not. Based on widefield functional optical imaging studies that have measured functional connectivity, if an anesthetic is needed, avertin or ketamine-xylazine is recommended. However, performing functional connectivity analysis in the awake state is optimal.

### 4.2 Neurovascular Coupling

In the normal brain, NVC refers to the local increase in CBF in response to neuronal activity.^106^^–^^108^ NVC is a critical control mechanism to supply sufficient nutrients to neurons during activation.^21^ In the context of widefield functional imaging in rodent models, NVC has been widely used to assess the degree to which the neurovascular response is coupled or uncoupled in diseased and injured brains. We discuss three stimulation paradigms used to assess NVC in rodents (1) hindpaw and forepaw stimulation, (2) whisker stimulation, and (3) visual stimulation. Although auditory stimulation^109^ has been performed to assess NVC, we do not discuss it in this review.

With electrical stimulation of the hindpaw or forepaw, several parameters can be modified to assess NVC. Modifiable parameters include the stimulation amplitude (current), pulse width, total stimulation duration, and frequency. The current delivered to the rodent paw ranges from 0.5 to 1.5 mA.^26^^,^^110^^–^^113^ A higher current will typically lead to a larger hemodynamic response. However, care must be taken to not exceed a current that causes pain, as this response differs from a traditional NVC response. Current above 1.5 mA may elicit a pain response and should not be used to study NVC dynamics.^114^ Other modifiable parameters also have variability. The pulse width in studies ranged from 300  μs to 1 ms, whereas the stimulation frequency can range from 1 to 10 Hz. Finally, the stimulation duration can vary from 2 to 60 s. Although these ranges are fairly large, the most common parameter set is an ∼1  mA current, a pulse width of 300  μs, a stimulation frequency of 3 Hz, and a stimulation duration of 2 s.^26^^,^^111^^,^^113^ One disadvantage to electrically stimulating the paw of rodents is that the animal needs to be under anesthesia. Studies have used a large variety of different anesthetics, such as isoflurane,^115^ alpha-chloralose,^26^ dexmedetomidine,^116^ medetomidine,^117^ and ketamine-xylazine.^112^ The type of anesthetic alters the NVC response to stimulation, so care should be taken when selecting an anesthetic in NVC studies.

Whisker stimulation is a standard method to assess NVC in rodents. Several different methods can be used to stimulate whiskers, such as using an air puff,^118^^,^^119^ a stepper motor with an attachment,^120^^,^^121^ or electrically stimulating the trigeminal nerve or whisker pad.^122^^,^^123^ Furthermore, a single whisker^124^^,^^125^ or multiple whiskers^126^ can also be used to assess NVC. Electrically stimulating the trigeminal nerve is similar to electrically stimulating the hindpaw and forepaw. Studies that have used electrical stimulation had a wide range of parameters. The parameters had the following ranges: current delivered was from 0.2 to 1.5 mA, pulse width was from 0.1 to 1 ms, stimulation frequency was from 0.25 to 40 Hz, and a stimulus duration from 4 to 30 s. As opposed to paw and whisker electrical stimulations, air puff and stepper motor methods are advantageous since imaging can be performed in awake rodents. Studies that used a stepper motor have an object attached to the motor to deflect the whisker in a repeatable manner. Stepper motor experiments modified the stimulation frequency and duration. Most studies use a frequency between 3 and 10 Hz with a duration between 2 and 20 s. Finally, the air puff modified the input settings for stimulus duration from 1 to 30 s, frequency from 3 to 5 Hz, and pulse duration from 1 to 100 ms.

Visual stimulation is advantageous such as whisker stimulation since anesthesia is not required to assess NVC. Many different methods exist to elicit an NVC response to visual stimulation. It is generally performed using a monitor that displays a spatial pattern of rotating bars on the screen. These spatial patterns can have different gray levels, degrees to how much they are rotated, different spatial frequencies, and display duration. The display of spatial patterns elicits an NVC response in the visual cortex, instead of the somatosensory cortex with other NVC methods.^127^^–^^129^

There are different analysis methods to quantify the NVC response. Two standard quantifiable metrics are the magnitude and rate of change in response to stimulation. The magnitude can be quantified as the peak response or the maximum change in CBF [Fig. 2(b)] or HbT. Furthermore, the rate of change is related to how fast the hemodynamic response is to stimulation, which can be calculated by taking the slope of the temporal hemodynamic data [Fig. 2(b)]. Using these quantifiable metrics, statistical tests can be performed comparing diseased animals with control animals or among the same animals over time.

### 4.3 Cerebrovascular Reactivity

CVR is the ability of cerebral vessels to dilate in response to vasoactive stimuli. CVR is widely used in humans to assess cerebrovascular health and function, such as in patients with cerebral amyloid angiopathy (CAA).^130^^,^^131^ With widefield functional optical imaging in rodents, there are two main approaches to assess CVR: (1) injection of acetazolamide or (2) gas inhalation. These approaches do not have as many knobs to turn as NVC approaches for the experimental setup. Acetazolamide increases ${\mathrm{pCO}}_{2}$ and acts as a potent vasodilator, which enables the study of CVR. Studies that use acetazolamide administer it intravenously with a concentration of 14 to 50  mg/kg.^132^^,^^133^ Acetazolamide has also been given intraperitoneal in awake mice with a concentration of 90  mg/kg.^134^ Since acetazolamide has a relatively long half-life, only the rise-time and maximum change in hemodynamics can be analyzed. Gas inhalation methods use ${\mathrm{CO}}_{2}$ to induce hypercapnia, which induces vasodilation. These experiments typically have a baseline period where the gas inhaled is under room air with isoflurane concentrations between 0.75% and 2%. After the baseline period, a period where the ${\mathrm{CO}}_{2}$ concentration is increased to a level between 5% and 10%, with the balance room air. The time under increased ${\mathrm{CO}}_{2}$ concentration ranges from 1 to 10 min.^135^^–^^138^ After this period, conditions are changed back to baseline conditions. The gas inhalation is advantageous compared with using acetazolamide because one can quantify what happens during the vasodilatory period of increased ${\mathrm{CO}}_{2}$ concentration and after the vasoactive stimulus is removed.

Similar to quantifying NVC dynamics, there are different methods to assess CVR. The magnitude can be described as the peak response or maximum change in CBF [Fig. 2(b)] or HbT. Furthermore, a rate of change can be calculated by taking the slope of the temporal hemodynamic data after the onset of the vasoactive stimuli. In addition, a rate of change can be analyzed as the time when the hemodynamic response reaches 50% of the peak response ($t_{50\;\text{rise}}$) or when the vasoactive stimulus is removed and the hemodynamic response decays, a $t_{50\;\text{fall}}$ can be obtained. Finally, the area under the curve (AUC) from the temporal hemodynamic data is a common metric that can be calculated during the hypercapnia period. These metrics allow for statistical tests to be performed that can compare diseased animals to control animals or among the same animals over time.

## 5 Applications

### 5.1 Optical Intrinsic Signal Imaging

**Fig. 4:**
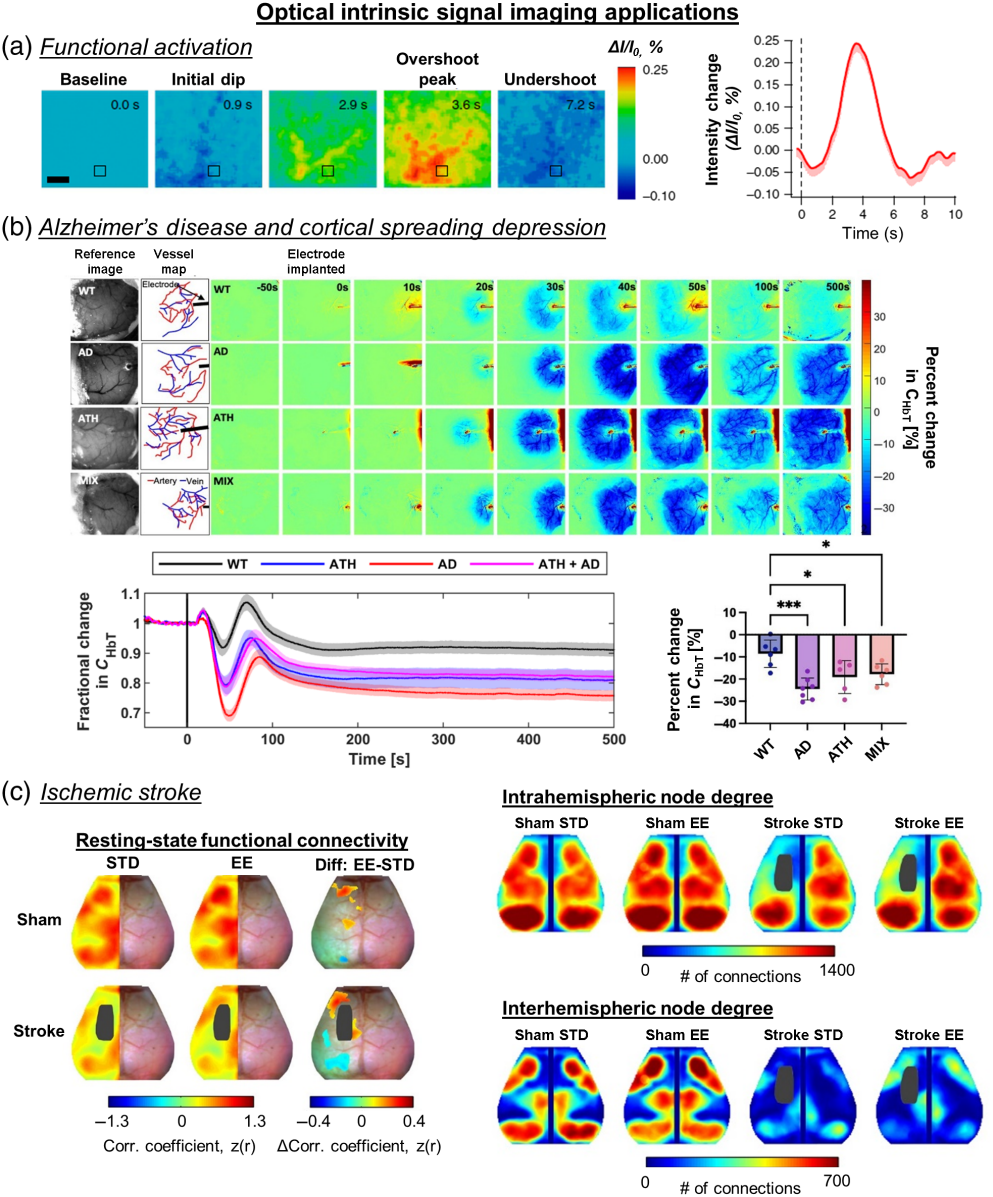
Representative applications of OISI in rodents. (a) A typical triphasic response at 630 nm using traditional OISI normalized to t=0  s. 630 nm is more sensitive to HbR concentration, which leads to an increase in intensity during the hyperemic phase. An “initial dip” is observed before the hyperemic peak followed by an undershoot (adopted from Ref. 144, with permission from Springer Nature). Scale bar = 0.5 mm. (b) (Top) Representative images of WT (wild-type), J20-AD (AD), PCSK9-ATH (atherosclerosis), and J20-PCSK9-MIX (AD + ATH) mice showing $C_{\mathrm{HbT}}$ changes postelectrode insertion. Electrode insertion occurs at t=0  s. The color bar represents the percent change in $C_{\mathrm{HbT}}$ from baseline. (Bottom left) Fractional changes in $C_{\mathrm{HbT}}$ in the different mouse groups. The black vertical line represents the time the electrode was inserted at t=0  s. (Bottom right) Quantitative differences of the percent change in $C_{\mathrm{HbT}}$ due to electrode insertion-induced CSD (adapted from Ref. 145). (c) (Left) Resting-state functional connectivity maps following sham or stroke in STD or EE housing. (Top right) Intrahemispheric and (bottom right) interhemispheric node degree averaged maps of sham and stroke groups after housing in STD or EE. Stroke diminished intrahemispheric node degree in the lesioned hemisphere and reduced interhemispheric node degree in all regions in both hemispheres compared with sham STD. Exposure to EE after stroke increased intrahemispheric and interhemispheric connections compared to STD housing (adapted from Ref. 146).

The first application of OISI in rodents measured the response of the somatosensory cortex to whisker stimulation.^40^ This paper reports one of the first studies to suggest an interconnection between neuronal activities and the surrounding vasculature. Early adaptations of OISI focused on exploring and understanding the neurovascular interactions in response to stimulations and identifying functional areas within the cortex. Expanding on the idea, Masino et al.^139^ utilized OISI to image the rat’s barrel cortex in response to whisker stimulations through a thinned-skull cranial window and noted a high correlation between OISI mapping and electroencephalogram (EEG) signals. In similar studies, the Frostig group^140^^–^^143^ noted a “triphasic” OISI signal in wavelengths that are sensitive to oxidation states of hemoglobin (e.g., 630 nm is more sensitive to HbR) during whisker stimulation in rats. For a 630-nm LED light source, the three phases in the OISI signal include (1) a slight decrease, (2) a significant increase, and (3) an undershoot leading to a return to baseline. Here, the early decrease in reflectance is referred to as an “initial dip,” where HbR increases while ${\mathrm{HbO}}_{2}$ decreases due to metabolic demands prior to resupply, which is often found in MRI literature [Fig. 4(a)].^144^^,^^147^ This “initial dip” is specifically prevalent at wavelengths that are sensitive to HbR, such as 630 nm [Fig. 4(a)]. However, it should also be noted that Lindauer et al.^148^ did not observe an “initial dip” after correcting for path length using optical properties. Thus, the presence of the “initial dip” remains up for debate.^147^ Combining spectroscopic OISI with LSI led to a more comprehensive view of hemodynamic activities in rats from whisker and forepaw stimulation to be obtained.^43^^,^^44^ The time course of CBF, ${\mathrm{HbO}}_{2}$, HbR, and cerebral blood volume (CBV obtained through a surrogate of HbT) were tracked. The study found that CBF, ${\mathrm{HbO}}_{2}$, and HbT increase following whisker stimulation, whereas HbR decreases, suggesting an increase in metabolic activities with a resupply of blood and oxygen from the surrounding vascular network. We next review OISI in applications of AD, CSD, and ischemic and hemorrhagic stroke.

#### 5.1.1 Alzheimer’s disease

OISI has been applied to study cerebral hemodynamics in AD and AD-related mouse models. For example, Bero et al.^149^ used OISI to study the difference between the APP/PS1 AD mouse model and wild-type mice through resting-state functional connectivity maps. The authors used ${\mathrm{HbO}}_{2}$ maps and seed-based correlation to generate coefficients of connectivity over the cortex. The study found that the presence of Aβ leads to disrupted bilateral functional connectivity. Specifically, this disruption, despite happening before Aβ formation, correlated with the amount of Aβ found later in life [Fig. 4(b)]. This finding supports the hypothesis that a CBF deficit and neurovascular dysregulation precede Aβ deposition.^150^ Another study found that exercise increased the peak ${\mathrm{HbO}}_{2}$ response to whisker stimulation in the APP/PS1 AD mouse model.^47^

Pericyte degeneration is associated with the development of dementia and AD. A recent study^150^ suggests that pericyte degeneration (Pdgfrb+/-) in mice leads to a deficit in NVC and oxygen supply in response to hind limb stimulation. Using original OISI, which relies only on reflectance changes, the researchers noted that the Pdgfrb +/- mice had a smaller decrease in reflectance at 530 nm and a smaller increase at 627 nm in response to hind limb stimulation. These reflectance changes indicate that the Pdgfrb +/- mice had a smaller increase in total blood volume and reduced oxygen delivery in response to hind limb stimulation. In a study with the J20-hAPP mouse model of AD, spectroscopic OISI was used to study hemodynamic activities between AD and wild-type mice under various stimulation conditions.^151^ The study found that there was no difference in hemodynamic activities between AD and WT mice during short and long durations of whisker stimulation and mild and major gas challenges with oxygen and ${\mathrm{CO}}_{2}$.^151^ Specifically, the study noted no difference in hemoglobin (i.e., ${\mathrm{HbO}}_{2}$, HbR, HbT) concentrations. However, the researchers noted a deficit in hemodynamic response to stimulations in young 6-month-old J20-hAPP mice when electrodes were introduced to measure electrophysiology of the same experimental conditions. Here, the authors attributed this discrepancy to CSDs caused by electrode implantation, which further exacerbated the already compromised neurovascular activities in the AD model. Using the same mouse model with a chronic cranial window preparation and microelectrode implants, showed that the AD mice had an increase in HbT (i.e., CBV increases) during a normobaric hyperoxia gas challenge.^152^

Further expanding on this study, an atherosclerosis model (PCSK9-ATH) was combined with J20-hAPP (AD) mice to examine cardiovascular comorbidities between 9 and 12 months of age.^145^ The researchers found that CSDs induced by the implanted electrodes led to prolonged hypoxia. Furthermore, the diseased mouse models (AD, ATH, and AD+ATH) had more severe CSD-induced vasoconstriction compared to wild-type mice [Fig. 4(b)].^145^ In another study, the researchers showed that the AD mouse model AβPPSWE/PS1ΔE9 developed a more significant increase in HbT and ${\mathrm{HbO}}_{2}$ concentrations in response to hindpaw stimulation at 7 months of age.^153^ The authors linked this increase in hemodynamic activity measured by OISI with larger vessel diameter changes measured using multiphoton microscopy during the stimulation time. These dynamics suggest hyperactivation during the early phases of AD pathogenesis, which has been seen with increased CBF during the early stages of AD development.^154^^,^^155^

#### 5.1.2 Cortical spreading depression

An important application of OISI is visualizing the spatial dynamics of CSD. In neurology, CSD is defined as a slowly propagating wave of depolarizing neurons and glial cells followed by a suppression of electrical brain activity that also leads to changes in vessel diameter, CBF, and metabolic activities.^156^ The presence of CSD is often related to worse clinical outcomes. The first study that used OISI to assess CSDs found highly asymmetrical and inhomogeneous spreading of the CSD wave in rats after potassium chloride (KCl) on the cortex.^157^ A multiwavelength OISI (i.e., 550, 610, and 850 nm) approach was applied to visualize and temporally track CSD from a pin prick rat model.^158^ The study found that there are three phases in the OISI signal for a region undergoing a CSD wave. Phase 1 includes an increase in reflectance at all wavelengths that the authors attributed to a change in the tissue scattering. Phase 2 can be separated into parenchymal (2p) and vascular (2v) areas. A decrease in reflectance at all wavelengths is observed in phase 2p due to blood volume increasing and cellular swelling decreasing. In phase 2v, the reflectance increases at 610 nm but decreases at 550 and 850 nm. This indicates that HbR decreases, but CBV increases, which leads to an oversaturation of the oxygen supply. Phase 3 shows the reflectance increasing at 550 and 850 nm, but decreasing at 610 nm, which corresponds to a decrease in CBV and an increase in HbR.

Following these initial hemodynamic findings, Brennan et al.^159^ utilized broadband (400 to 700 nm) OISI to track CSD in mice and rats. The study found that the propagation speed is slower in mice than in rats, and mice have a larger second-phase amplitude. The increase in signal amplitude during the second phase is associated with mice having a greater hypoperfusion period. A follow-up study found that female mice have a lower threshold to induce CSD using KCl and tetanic stimulation.^160^ Spectroscopic OISI was applied to monitor the hemodynamics in a rat brain following a pin-prick CSD induction.^43^ The study found an increase in CBV, measured using HbT as a surrogate, along the propagation of the CSD wave, which agrees with previous studies that show a decrease in all signals during phase 2p (parenchymal). Subsequent studies examined CSD induced by KCl in mice and noted a decrease in CBV instead of a normal hyperemic response in rats attributed to vasoconstriction,^161^ which agrees with a decrease in oxygenation that is characteristic of hypoxia.^159^ Aside from direct induction of CSD using pinpricks, tetanic, and chemical solutions, others have studied CSD associated with other brain diseases, including stroke, which we discuss below.

#### 5.1.3 Ischemic and hemorrhagic stroke

Most published OISI stroke studies focus on focal events and the subsequent hemodynamics. Chen et al.^162^^,^^163^ used OISI to study middle cerebral artery occlusion (MCAO)-induced CSDs in rats. They showed that ischemia-induced CSDs had a diffuse response compared to that following a pinprick stimulus. The wavefront of the CSDs caused by MCAO did not possess the usual well-defined circular shape as that of a pin-prick response. Interestingly, the origin of the CSDs was determined to be within the penumbra, not the stroke core. Furthermore, the study showed that the reflectance increased in the penumbra due to hypoperfusion and decreased in normal areas due to hyperperfusion, which could be attributed to a compensatory mechanism following MCAO. Jones et al.^164^ utilized LSI and spectroscopic OISI to track hemodynamic responses following a distal MCAO (dMCAO) in mice. Specifically, the study noted that the modified Beer–Lambert’s law usually used in OISI processing overestimated HbR and underestimated ${\mathrm{HbO}}_{2}$ following stroke. Due to this discrepancy, the authors derived a new method to perform nonlinear fitting of reflectance from a Monte Carlo photon migration model for $\mu_{a}$ and $\mu_{s}^{\prime }$ values to obtain chromophore concentrations. The study found that ${\mathrm{HbO}}_{2}$ decreased while HbR increased, which signifies that blood and deoxygenation of blood become stagnant after dMCAO. The authors also visualized anoxic depolarization as a secondary reduction in all hemodynamic parameters, and peri-infarct depolarizations (PIDs) that occurred sporadically as all hemodynamic parameters decreased except HbR. Using spectroscopic OISI and calcium indicator (GCaMP) imaging to monitor CSDs following photothrombosis revealed that successive CSDs led to more tissue damage beyond the initial insult and altered the neurovascular response to subsequent CSDs.^45^ The regions with lower baseline perfusion were subject to more vasoconstriction from subsequent CSDs that exacerbated metabolic stress and cortical damage.

Researchers have also used OISI to study neural plasticity and the functional reorganization of the brain following a focal ischemic stroke. For example, photothrombotic stroke led to a remapping of the functional regions within the motor cortex.^165^ The remapped functional regions had a more diffuse pattern than the response following forelimb and hindlimb stimulations.^165^^,^^166^ Bauer et al.^167^ used OISI to study functional connectivity in mice undergoing transient MCAO (tMCAO). Specifically, the authors used the spatial mapping of ${\mathrm{HbO}}_{2}$ at several time points to generate a functional connectivity map. The study found that functional connectivity declined with the severity (e.g., time length) of tMCAO and also impaired CVR to stimulus in the somatosensory cortex. Other studies have used OISI to track the response of specific treatments or rehabilitative methods following ischemic stroke. OISI was used to measure resting-state functional connectivity maps following sham or stroke in standard environment (STD) or enriched environment (EE) housing.^146^ Sham mice housed in EE had enhanced resting-state functional connectivity compared with sham STD mice in parts of the frontal, secondary motor, and posterior primary motor areas. Stroke mice had reduced resting-state functional connectivity in STD and EE groups. There were also differences in the resting-state functional connectivity strength in motor regions between stroke STD and stroke EE groups. Furthermore, the number of connections (degree) of each pixel (node) was determined by thresholding the resting-state functional connectivity maps at z(r)≥0.4. Summing the correlation coefficients above this threshold produced a measure of intrahemispheric and interhemispheric node degree.^146^ Stroke diminished intrahemispheric node degree in the lesioned hemisphere and reduced interhemispheric node degree in all regions in both hemispheres compared with sham STD. Exposure to EE after stroke increased intrahemispheric and interhemispheric connections compared with STD housing [Fig. 4(c)].^146^ EE or naturalistic habitation promotes the expression of natural, innate behaviors and alters cortical sensory maps.^168^ OISI was used to determine that mild stimulation immediately after a permanent MCAO allows for activation and protection of some cortical functions,^169^^,^^170^ whereas mice treated with memantine after a photothrombotic stroke had a larger and more focused (less diffuse) sensory map.^171^

OISI was recently applied to track CSDs and hemodynamics following hemorrhagic stroke. Specifically, OISI was used to track functional connectivity in mice immediately and 3 months after subarachnoid hemorrhage (SAH) occurred.^172^ The study found seed-based connectivity decreased in the motor, retrosplenial, and visual cortex, lasting up to 3 months. Furthermore, following SAH, decreased CBF and ${\mathrm{HbO}}_{2}$ were observed after a KCl-induced CSD, which correlated with a lower neurological score.^173^

### 5.2 Laser Speckle Imaging

LSI has been widely applied to rodents for many neurologically relevant applications. This section reviews LSI applications to AD, focal stroke, cardiac arrest and resuscitation, and traumatic brain injury (TBI).

#### 5.2.1 Alzheimer’s disease

LSI has been used in studies of different mouse models of AD to examine resting-state and dynamic CBF. In addition to AD, LSI has been used to investigate risk factors for AD, such as aging,^137^ genetic factors such as APOE4,^174^ and environmental factors, such as hypertension.^175^ We do not discuss these risk factors in this review, but they are important to consider in the context of AD. A large portion of studies that include LSI are used to provide supporting information on cerebrovascular function in mechanistic studies of AD.

**Fig. 5:**
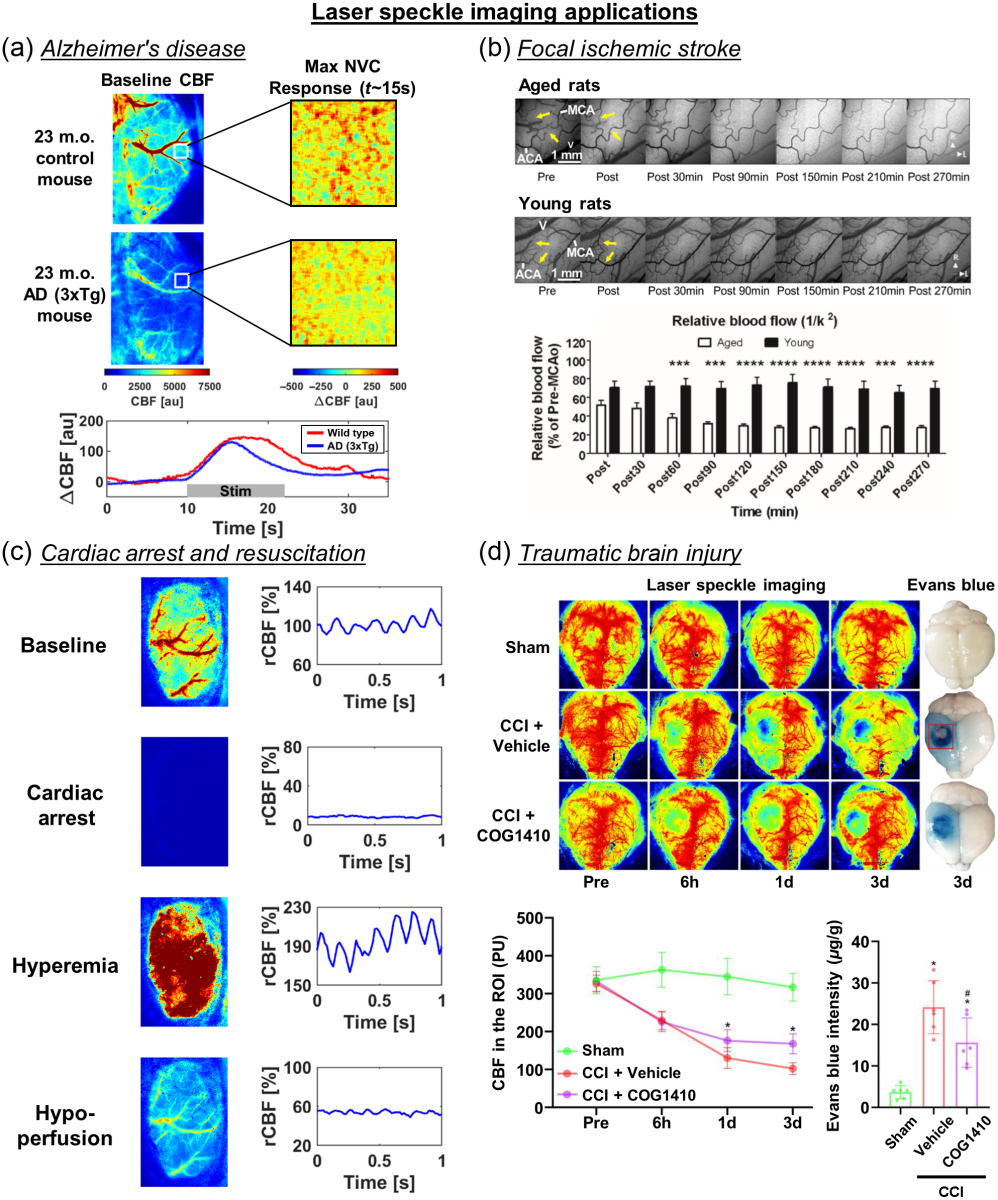
Representative applications of LSI in rodents. (a) Representative data showing that a 23-month-old AD (3xTg) mouse has lower baseline CBF (left) and impaired NVC response (right and bottom) compared with an age-matched control mouse. (b) Visualization of CBF in the penumbra after MCAO induced focal ischemia in aged (top row) and young (middle row) rats, and time trace comparisons (bottom row) (adapted from Ref. 181). (c) Asphyxial cardiac arrest followed by CPR reveals distinct overall and pulsatile CBF phases throughout a cardiac arrest and resuscitation experiment (baseline, cardiac arrest, hyperemia, and hypoperfusion). (d) A TREM2 agonist, COG1410, improved CBF (top and bottom left) and mitigated BBB breakdown (top and bottom right) following CCI-induced TBI (adapted from Ref. 182).

Tachibana et al.^176^ implanted pericytes into one brain hemisphere in 18- to 20-month-old APP/PS1 mice. Three weeks after implantation, the resting-state CBF was measured with LSI over the whole cortex. Interestingly, the CBF was higher on the ipsilateral side of pericyte implantation compared with the contralateral side, which coincided with significantly lower insoluble Aβ40 and 42 and Aβ plaque burden. This work agrees with clinical studies that observe lower CBF in AD patients.^177^^,^^178^ Furthermore, Aβ oligomers have been shown to produce capillary vasoconstriction via pericyte signaling.^179^ This is expected to cause a global reduction in CBF since capillaries are the most common blood vessel in the brain.^150^ Interestingly, 9-month-old 3xTg male mice showed a reduction in cortical CBF, which corresponded with decreased CD31 (vascular marker) and increased Aβ labeling.^180^ Our data from 23-month-old 3xTg mice also support reduced cortical CBF [Fig. 5(a)]. However, recent comprehensive genotyping and phenotyping of the 3xTg model suggests that the pathological development of Aβ plaques, and other AD characteristics has drifted compared with the originally developed 3xTg model.^183^

Shin et al.^135^ assessed CVR and NVC in response to hypercapnia and whisker stimulation, respectively, using Tg2576 mice at 8- and 19-months old. Previous publications showed that cerebrovascular Aβ, or CAA, begins in Tg2576 mice around 9-months old, with severe CAA by 18-months old. This study showed that Tg2576 mice had impaired CVR and NVC at 19-months old, but not at 8-months old, compared with wild-type control mice. These results suggested that vascular Aβ was necessary to impair cerebrovascular function. However, this paper did not report histological analysis to assess the degree of CAA at 8- and 19-months old. Furthermore, there were trends toward impaired CVR and NVC in both wild-type and Tg2576 with increasing age. Subsequently, Tarantini et al.^63^ used a commercial LSI system to measure NVC using a whisker stimulation protocol on 11-month-old Tg2576 mice. Similar to previous studies, they showed reduced NVC in the Tg2576 mice compared with age-matched control mice. Furthermore, we show that 23-month-old 3xTg mice have impaired NVC [Fig. 5(a)].

Several published studies have examined the association between Aβ and cerebrovascular changes. However, the association between tau and cerebrovascular changes is less studied. Park et al.^184^ used LSI to investigate the neurovascular changes in two transgenic tauopathy mouse lines, rTg4510 and PS19. This study showed that young (2 to 3 months old) transgenic mice had worse NVC than wild-type controls before the onset of neurofibrillary tangles and cognitive impairment. Interestingly, turning off tau production with doxycycline reversed neurovascular dysfunction. A separate study also showed that the resting-state CBF was lower in PS19 mice than WT controls at 12-months old.^185^ These rodent findings have also been seen in AD patients with tau pathology.^186^

#### 5.2.2 Focal ischemia

Focal ischemia is one of the most prominent applications of LSI. Unlike AD studies, focal ischemic events produce real-time CBF alterations, making LSI a compelling technology for visualizing CBF dynamics in the ischemic core, penumbra, and nonischemic tissue. The initial study^187^ visualized CBF dynamics in response to a distal middle cerebral artery (MCA) ligation. The study quantified CBF changes in the core, penumbra, and nonischemic tissue. There was a decrease of ∼80% in the core and 50% in the penumbra.^187^ Subsequent studies that used LSI to monitor focal ischemia analyzed the relationships between CBF and electrophysiological, histological, and molecular changes.

Studies show that decreased CBF due to focal ischemia results in spreading depolarizations (SDs). These studies have visualized these dynamics due to simultaneously using LSI and electrophysiological techniques to detect SDs. Furthermore, sustained ischemia and reduced CBF caused repeated PIDs. A corresponding decrease in CBF would occur in the core and penumbra with each PID.^164^^,^^188^ Multiple PIDs worsen neurological injury in both animal models^189^^,^^190^ and humans.^191^ A greater decrease in CBF due to focal ischemia-induced SD was mitigated through an NMDA antagonist MK-801 and resulted in minimizing the infarct tissue volume.^188^ Interestingly, inducing hypertension after dMCAO lessened infarct volume and increased the reduction in CBF due to PID.^192^ Furthermore, to assess focal ischemic stroke recovery, studies have combined MCAO with comorbidities, such as hyperlipidemia. One study examined the integrin α9β1, which is highly expressed on activated neutrophils, in a comorbid model of hyperlipidemia. The study found that CBF recovered faster in α9-deficient mice with hyperlipidemia compared with hyperlipidemia mice alone. The CBF recovery coincided with reduced fibrin, platelet thrombi, neutrophil, and decreased tumor necrosis factor-α and IL (interleukin)-1β levels.^193^ Finally, penumbral CBF recovery following MCAO varied with age [Fig. 5(b)].^181^ Aged rats had a decrease in CBF to 40% of baseline 4.5 h following MCAO, whereas young rats had a CBF of 60% to 80% of baseline. This study showed that although cerebral collateral perfusion declined for both young and aged rats, aged rats were more affected.

Another method to induce focal ischemia is an intravenous injection of Rose Bengal and excitation with laser light to induce local ischemic events. This method can target specific regions of vessels or even a single vessel.^194^ In addition, LSI has been used to study the spatiotemporal CBF changes of a single vessel that undergoes photothrombosis. Sullender et al.^195^ revealed that a targeted ischemic event on a penetrating arteriole leads to a decrease in both the targeted vessel and the surrounding vascular region. Furthermore, longitudinal assessment with LSI revealed reperfusion of the targeted arteriole ∼1 week after the ischemic event in addition to the oxygen tension returning to near baseline levels.^195^ A subsequent study simultaneously measured CBF and the local field potential (LFP) response to a single-vessel photothrombotic event. Although the CBF response returned to baseline levels, there was a distinct neurovascular dissociation due to a prolonged reduction in LFP. This study demonstrates that caution should be used when using hemodynamic information to infer neural activity.^196^

#### 5.2.3 Cardiac arrest and resuscitation

A rapid decrease in CBF occurs when entering CA via asphyxiation, ventricular fibrillation, or intravenous administration of KCl. CA induces global ischemia in the brain due to inadequate blood flow from the heart. Resuscitation of a subject typically requires the administration of cardiopulmonary resuscitation (CPR) and drugs such as epinephrine and sodium bicarbonate. Upon successful resuscitation, blood circulates throughout the body and importantly, the brain. LSI has been used to monitor CBF dynamics in CA and resuscitation models in largely proof-of-concept studies. Due to the high spatiotemporal resolution of LSI, CBF dynamics in multiple regions of interest (ROIs) can be analyzed to monitor cortical CBF, arterial and venous CBF, and parenchymal CBF, in addition to pulsatile CBF. We discuss studies that show distinct CBF characteristics associated with entering CA,^197^^,^^198^ postresuscitation hyperemia, and sustained hypoperfusion period^61^^,^^199^ [Fig. 5(c)].

A rapid decrease in CBF occurs immediately after the onset of CA. Approximately 30 s after the onset of asphyxial or KCl administration, there was a slight rebound in the CBF, which may be due to increased blood pressure during this time^61^^,^^197^ and potentially related to the onset of EEG silence or increased EEG coherence.^200^ After ∼2  min of CA, a wave of SD occurs, similar to focal ischemia. This wave coincides with a negative deflection in the DC potential, increasing cerebrovascular resistance, and a wave of decreasing CBF.^198^ In the immediate post-CPR period, there is a strong hyperemia period. Interestingly, this period has been shown to have a no-reflow phenomenon where red blood cells stall in capillaries.^201^ We showed that the total amount of CBF during the post-CPR hyperemia period is directly related to the initial EEG burst, which is the first-moment brain electrical activity starts after CA and resuscitation. We were able to develop a mathematical model to predict the initial EEG burst.^61^ The onset of the initial EEG burst coincided with the start of a prolonged hypoperfusion period. The onset of the initial EEG burst and prolonged hypoperfusion periods were more delayed with increased CA severity. Others have shown that target temperature management using therapeutic hypothermia increased the CBF response in the post-CPR period and improved neurological recovery.^60^^,^^202^ Furthermore, vagus nerve stimulation during the postresuscitation period increased CBF and better neurological recovery from histological and behavioral scores.^203^^,^^204^ Examining pulsatile CBF with LSI during CA and resuscitation revealed that the pulsatile blood flow changed similarly to overall CBF.^62^ During the hyperemia phase, there was increased CBF pulsatility, whereas during the hypoperfusion period there was decreased CBF pulsatility. However, these changes in CBF pulsatility did not correlate with short-term EEG recovery, whereas increased peripheral blood pressure during the hypoperfusion period was associated with worse EEG recovery.^62^

#### 5.2.4 Traumatic brain injury

LSI has been used to study the spatiotemporal CBF changes that occur in different models of TBI, such as controlled cortical impact (CCI) for more severe TBI and closed head injury for mild TBI. The initial CBF response due to mild TBI resulted in a wave of decreasing CBF from the impact site that propagated at a rate of 3.5  mm/min.^205^ This SD wave is also seen in focal and global ischemia. The SD wave induced an oligemia or CBF deficit period that was sustained for 90 min after the closed head injury, at which time the CBF returned to baseline levels. Another study^206^ used LSI at two different wavelengths to demonstrate that CBF decreased following TBI in a similar model and validated this result against laser Doppler flowmetry. Furthermore, LSI was used to measure hypercapnia-induced CVR 3 and 6 months after a period of repeated mTBI that lasted 3 months. The AUC of the CBF during hypercapnia was measured proximally and distally from the injury site. Interestingly, at 3-months after the last mTBI, both proximal and distal ROIs had a lower AUC of the CBF during hypercapnia, whereas at 9-months after the last mTBI, only the proximal ROI had a lower AUC of the CBF during hypercapnia.^38^ Furthermore, CCI-induced TBI resulted in a CBF reduction that coincided with reduced vascular density.^207^ Notably, downregulating miR-491-5p, which is important in cell proliferation and migration, resulted in improved CBF, increased vascular density, better neurological function, and better survival of neurons after TBI. In addition, TREM2 impacts the extent of injury following CCI-induced TBI. TREM2 is an important transmembrane receptor primarily expressed on microglia and has implications in neurodegenerative diseases, such as AD.^208^ In a recent study, following CCI-induced TBI, a TREM2 agonist, COG1410, improved CBF, lessened BBB breakdown [Fig. 5(d)], improved neurological functions, and inhibited neutrophil infiltration and microglial activation. Importantly, using a TREM2 knockout mouse model reversed these benefits, suggesting that TREM2 is critical for protecting against TBI-related damage.^182^ These studies suggest that LSI can serve as a tool to better understand the hemodynamic response after TBI-induced CBF changes.

### 5.3 Spatial Frequency Domain Imaging

Applications of SFDI to neuroimaging are a more recent development. Here, we discuss SFDI in applications of AD, focal ischemia, cardiac arrest and resuscitation, and TBI.

#### 5.3.1 Alzheimer’s disease

Recently, multiple studies were performed using SFDI to measure brain optical properties in several mouse models of AD and neurodegeneration. The initial study^209^ employed SFDI at 17 discrete wavelengths from 650 to 970 nm to image the brains (through an intact skull) of 20-month-old triple-transgenic (3xTg) AD mice and controls. This study found that $\mu_{s}^{\prime }$ was ∼20% higher in the AD mice, the tissue water fraction was ∼70% higher in the AD mice, and $C_{\mathrm{HbT}}$ and ${\mathrm{StO}}_{2}$ were lower in the AD mice. Furthermore, AD mice exhibited a slower and 50% smaller hemodynamic response to an oxygen inhalation challenge compared with control mice. This study established the viability of SFDI as a means to quantitatively measure baseline and dynamic changes in tissue optical properties related to brain physiology in a rodent model of AD. A follow-up study^110^ used a three-wavelength visible-light SFDI system to image 20-month-old 3xTg mice. Similar to the previous study, this study found a higher $\mu_{s}^{\prime }$ in 3xTg mice. Furthermore, a differential path length factor was calculated to correct the hemodynamic data, yielding an increase in ${\mathrm{HbO}}_{2}$ due to hindpaw stimulation that was larger and longer in controls compared with AD mice.

**Fig. 6:**
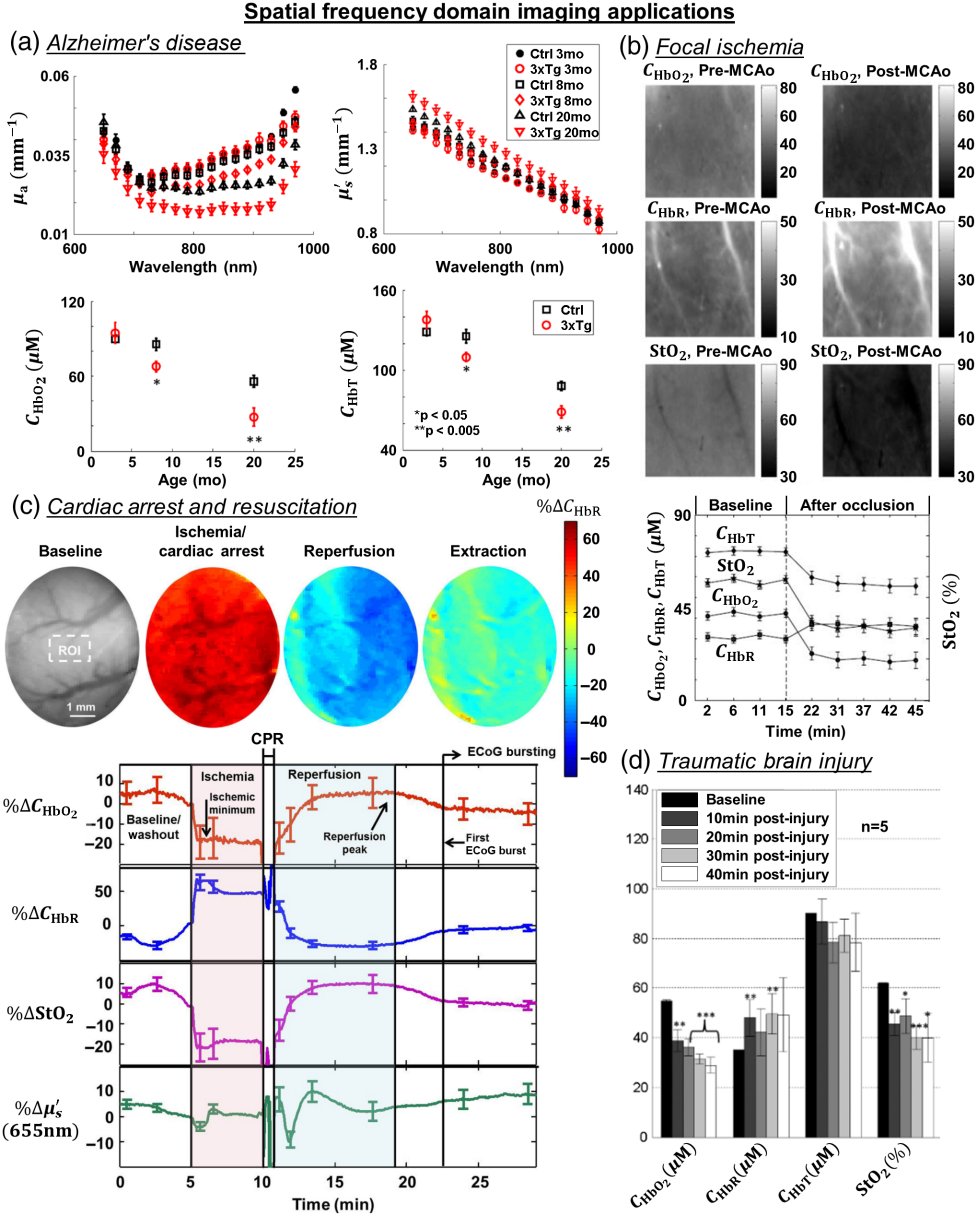
Representative applications of SFDI in rodents. (a) $\mu_{a}$ (top, left), $\mu_{s}^{\prime }$ (top, right), and hemoglobin concentrations (bottom) are altered in 3xTg AD mice by 8 months of age (adapted from Ref. 210). (b) The onset of focal ischemia reveals alterations in hemodynamic properties of $C_{\mathrm{HbO}2}$, $C_{\mathrm{HbR}}$, $C_{\mathrm{HbT}}$, and ${\mathrm{StO}}_{2}$ (adapted from Ref. 211). (c) Asphyxial cardiac arrest followed by CPR reveals distinct changes in ${\mathrm{StO}}_{2}$ and scattering in different phases of a cardiac arrest and resuscitation experiment (baseline, cardiac arrest, hyperemia, and hypoperfusion) (adapted from Ref. 76). (d) TBI-induced changes in $C_{\mathrm{HbO}2}$, $C_{\mathrm{HbR}}$, and ${\mathrm{StO}}_{2}$ but not $C_{\mathrm{HbT}}$ (adapted from Ref. 212).

Two additional studies aimed to interrogate physiological mechanisms that underlie tissue optical property changes in AD mice. One study^115^ imaged 3-month-old mice with transgenes for doxycycline-regulated neuronal expression of diphtheria toxin (CaM/Tet-DTa). Removing doxycycline from the diet resulted in neurodegeneration and a concomitant ∼15% increase in $\mu_{s}^{\prime }$ in the brain. This study provided evidence that neurodegeneration could lead to measurably higher values of $\mu_{s}^{\prime }$, which supports the hypothesis that $\mu_{s}^{\prime }$ changes could be attributed to AD-related neurodegeneration.^209^ The second study^210^ showed that in addition to optical property differences, a drop in brain tissue hemoglobin concentration in 3xTg mice occurred as early as 8 months of age [Fig. 6(a)] and coincided with vessel volume and density reduction along with increased Aβ load in the brain. Although $\mu_{s}^{\prime }$ has been shown to be a strong indicator of AD and neurodegeneration, the cellular mechanisms associated with these changes are difficult to elucidate. Due in part to this challenge, the use of SFDI in mechanistic studies has been limited.

#### 5.3.2 Focal ischemia

SFDI has also been employed to assess hemodynamic changes in the brain during a focal stroke. One study^211^ in an MCAO model in rats measured substantial decreases in ${\mathrm{StO}}_{2}$ and $C_{\mathrm{HbT}}$ in an imaging window over the left parietal somatosensory cortex [Fig. 6(b)]. A more recent study^213^ validated the use of tissue scattering measured with optical coherence tomography (OCT) for identifying brain regions damaged by stroke.

#### 5.3.3 Cardiac arrest and resuscitation

Recently, SFDI has been used in a series of studies to characterize hemodynamics and metabolism in response to cardiac arrest and resuscitation. In these studies, an SFDI imaging system was designed for the rodent brain to enable continuous, noncontact monitoring of brain tissue optical properties, hemoglobin concentrations, and oxygenation. This monitoring was performed during entry into cardiac arrest, CPR, and reperfusion in a setup mimicking an intensive care unit.^61^^,^^76^^,^^200^^,^^214^ This setup provided high-speed (∼14  Hz) SFDI mapping of chromophores, which enabled the requisite temporal resolution to monitor rapid hemodynamic changes and calculate cerebral pulsatility. SFDI revealed rapid cerebral changes in the hemoglobin concentrations and oxygenation during cardiac arrest and immediately post-CPR that were distinct from changes in the tissue $\mu_{s}$ during these same time periods [Fig. 6(c)].^76^ This finding illustrated the ability of SFDI to independently quantify dynamics in both tissue absorption and scattering during periods when both properties were rapidly changing. Furthermore, the combination of LSI, SFDI, and a “zero flow” calibration condition^215^ enabled the calculation of the ${\mathrm{CMRO}}_{2}$ in absolute physiological units ($\mu MO_{2}/\min$).^77^

#### 5.3.4 Traumatic brain injury

Over the past decade, SFDI has been used in studies to quantify cerebral hemodynamics and metabolic changes in rodent models of TBI. The first of these studies^212^ used SFDI to monitor cerebral absorption and scattering parameters in response to a weight-drop, closed-head-injury model in mice. The study observed a significant decrease in brain ${\mathrm{StO}}_{2}$ following the injury [Fig. 6(d)]. A subsequent study^216^ used a similar model to evaluate the effect of different pharmaceuticals used in hospital settings to treat TBI. An additional study^217^ compared the two-wavelength LSI approach with SFDI to monitor cerebral hemodynamics and metabolism in a similar model. Both techniques detected decreased CBF, oxygenation, and metabolism within the first hour following TBI.

## 6 Conclusions and Future Directions

The complex cerebrovascular dynamics that occur during AD and neurological injury highlight the importance of investigating cerebral hemodynamics. Widefield optical imaging approaches, such as OISI, LSI, and SFDI, have contributed to advancing our scientific understanding of hemodynamic changes in AD and neurological injury. To help elucidate mechanistic biological processes, widefield optical imaging has been combined with histological, molecular, biochemical, or electrophysiological analyses. Combining multiple approaches can enable the analysis of various physiological processes from a multiscale and functional perspective.

Advances in instrumentation and data analysis algorithms for widefield optical imaging approaches may provide additional hemodynamic information content. One additional hemodynamic measurement that can provide valuable information is the ${\mathrm{CMRO}}_{2}$ consumption. Previous studies have measured ${\mathrm{CMRO}}_{2}$ with the combination of LSI and OISI^43^^,^^44^ or SFDI.^77^^,^^215^ However, these measurements of ${\mathrm{CMRO}}_{2}$ have historically been on a relative scale. A recent advance is the combination of LSI and SFDI approaches to enable the absolute measurement of ${\mathrm{CMRO}}_{2}$. However, this approach requires a time period with a “zero-flow” condition to quantify the extraction of oxygen from hemoglobin on an absolute physiological scale (e.g., $\mu MO_{2}/\min$). Therefore, a method with minimal to no perturbation that measures absolute ${\mathrm{CMRO}}_{2}$ would provide significant advantages. This will enable baseline comparisons in metabolic activity between subjects or longitudinally track a subject over time. Since widefield functional optical imaging currently has relatively poor spatial resolution, increasing the spatial resolution over the entire cortex to assess blood flow, oxygenation, and metabolism at the NVU level would potentially be a transformative development. These advancements could play a major role in informing our biological understanding of diseased and healthy brains over microscopic and mesoscopic scales simultaneously.

Although this review only discussed OISI, LSI, and SFDI for the measurement of hemodynamic processes in the brain, there are several technologies that we did not highlight. For example, hyperspectral imaging, similar to OISI, employs a relatively large number of wavelengths, enabling a more accurate determination of chromophore concentrations. The increased spectral resolution can help better resolve chromophore concentrations in the tissue.^218^ In addition, OCT is another approach that enables an exquisite anatomical view of the vasculature with OCT angiography and functional blood flow information with Doppler OCT.^219^^–^^221^ Photoacoustic tomography (PAT) is an approach that can perform measurements that range from microscopic to macroscopic FOVs. Furthermore, PAT can measure oxygenation, blood flow, and metabolism changes.^222^^,^^223^ In addition, advances in multiphoton microscopy have enabled imaging over an ∼5  mm diameter,^224^ ∼7  mm diameter,^225^ and across hemispheres in mice.^226^ These advances enable high-resolution images of the vasculature and blood flow information of single vessels using line scans. Advances to widefield optical imaging approaches and multimodal instrumentation can enrich hemodynamic information content across spatial scales, help elucidate cerebrovascular mechanisms, and may lead to the development of therapeutic agents for AD and neurological injury.
